# On being better than average in values

**DOI:** 10.1111/bjso.70089

**Published:** 2026-04-28

**Authors:** Andrey Elster, Anat Bardi, Joanne Sneddon, Lilach Sagiv, Sonia Roccas, Julie Anne Lee

**Affiliations:** ^1^ Education and Psychology The Open University of Israel Ra'anana Israel; ^2^ Department of Psychology Royal Holloway, University of London Egham UK; ^3^ Business School University of Western Australia Perth Western Australia Australia; ^4^ Hebrew University Business School The Hebrew University of Jerusalem Jerusalem Israel

**Keywords:** better than average effect, personal values, self‐enhancement bias, self‐esteem

## Abstract

Do people feel that their personal values are better than others, even though they are happier if their values are similar to those around them? We examined the Better Than Average (BTA) effect in values in four cultures (Study 1: USA, China and Malaysia; Study 2: USA, using diverse online panel samples) and relative to either a more abstract (university) or a more concrete (department) reference group (Study 3, conducted with students in Israel). Across all samples and cultures, we found that people perceived their personally desired values as more important to the self than to others, and they perceived their less personally desired values as more important to others than to the self. Self‐other comparisons favouring the self were even stronger for values that are normatively desired in society, and self‐other comparisons favouring others were even stronger for values that are less normatively desired in society. We also found a relatively greater BTA effect towards a more abstract group and its positive consequences to self‐esteem. This research contributes to the theoretical understanding of value perception as prone to biases, generalizability and robustness of the BTA effect, and cross‐cultural psychology. We discuss important societal implications of this effect.

## INTRODUCTION

Do people see themselves as having better values than others? On the one hand, much research has found that people tend to see themselves as better than others in important competencies and positive traits (Krueger & Wright, 2011; Zell et al., 2020). Therefore, we may expect that people also see themselves as having better values than others. On the other hand, there is extensive research (reviewed in Sagiv & Schwartz, 2022) showing that congruency in values with one's environment is associated with well‐being. Hence, it is possible that people do not see themselves as having better values than others, because they may wish to see themselves as similar to others in their values. In this paper, we aim to shed light on these contradicting expectations by testing if people tend to see themselves as better than average (BTA) in values.

This aim is important for developing theoretical understanding of values and the BTA effect. Moreover, finding out whether people see their values as better than those of others has important societal implications as perception of others' values is meaningful in group contexts (López‐Rodríguez et al., 2023; Wolf et al., 2021), and has even been found to affect hope in inter‐group conflict (Wolf & Hanel, 2024). Thus, we examine whether such biased perceptions are employed on personally desired values and on values that are normatively desired in society, testing hypotheses with three studies, including a cross‐cultural comparison. We also test whether this effect is greater for perceptions of a more abstract reference group than for a more familiar group, as has been found regarding traits (Krueger & Wright, 2011). Finally, we examine a possible consequence of the BTA effect, namely self‐esteem. Past research indicated that perceiving oneself as BTA on personality traits increased self‐esteem (see Zell et al., 2020). This effect has not been examined yet with regard to values.

## THE BETTER THAN AVERAGE EFFECT

The BTA effect occurs when people view themselves as higher than others on desirable attributes and lower than others on undesirable attributes, usually compared with an average peer (Alicke, 1985). The BTA effect is a type of self‐enhancement bias (see Krueger & Wright, 2011) of perceiving oneself to have better attributes than others. These biases reflect a fundamental need for people to maintain a positive self‐regard. Such biases have been mostly associated with positive psychological, physiological and social outcomes, including higher self‐esteem (Zell et al., 2020) and better mental and physiological health (Dufner et al., 2019).

Research has documented the BTA effect in a wide range of studies across different traits, abilities and behaviours (Alicke & Govorun, 2005; Zell et al., 2020). Overall, this bias was found to be strongest in attributes that are subjective (Alicke et al., 1995), abstract (Dunning et al., 1989), moral (Dunning et al., 2005), desirable in society (Brown, 2012) and personally important (Alicke, 1985). The definition of values incorporates all these factors (Schwartz, 1992), suggesting that the BTA effect may occur also in personal values.

### Personal values

Personal values are defined as broad life‐goals varying in importance that serve as guiding principles in individuals' lives (Schwartz, 1992). They influence how individuals interpret their world (Schwartz et al., 2000). Values serve as standards for what is desirable and right, and as justifications for actions and opinions (Maio, 2010). Ample research has documented the systematic associations of values to many attitudes (Boer & Fischer, 2013), behaviours (Sagiv & Roccas, 2021), emotions (Tamir et al., 2016) and cognitive biases (Elster & Sagiv, 2025). Values operate as a system, as multiple values are likely to be relevant to the same situation; hence, behaviour or opinion is likely determined not by the raw importance of the value but by prioritizing a value over one's other values (Schwartz, 2012).

In this paper, we use the most empirically established theory of values—the Schwartz (1992) values theory. This theory specifies 10 broad values, structured in a circle of relations, in which each value is most positively correlated with neighbouring values in the circle and most negatively correlated with opposite values in the circle (for more details, see Supporting Information). The 10 broad values and their circular structure have been validated in numerous countries worldwide (Schwartz, 2015).

People refer to values when explaining their choices and actions, and when evaluating others (Maio, 2010). While self‐reports of values are probably genuine, showing little evidence for social desirability or impression management (Bye et al., 2011; Danioni & Barni, 2021; Schwartz et al., 1997), inferences about the values of other people may be systematically biased, especially when assessing values of distant others (Hanel et al., 2018). Research has found that individuals are quite accurate in their perceptions of the values of close others, like friends and family members (Bernard et al., 2006; Dobewall et al., 2014), but misperceive the values of distant others belonging to broad social groups (Hanel et al., 2018). When making inferences about others, people tend to use their own attributes as a reference point, especially in abstract and subjective domains (Dunning et al., 2004), like values, enabling the BTA effect.

## BTA IN VALUES

Research on BTA identified several factors that amplify it, all relevant to values, rendering them susceptible to its effects. Specifically, the BTA effect was stronger for *subjective* attributes that lack measurable criteria than for attributes that can be objectively evaluated (Alicke et al., 1995; Van Lange & Sedikides, 1998). Values are subjective by definition, reflecting individually important principles (Schwartz, 1992). Furthermore, the BTA effect was stronger for relatively *abstract* attributes reflected in many behaviours than for relatively concrete attributes only relevant to specific behaviours (Dunning et al., 1989). Values are broad goals, affecting many behaviours across contexts (Bardi & Schwartz, 2003). Researchers also found that the BTA effect is larger for *moral* than for non‐moral attributes (Sedikides & Gregg, 2008; Tappin & McKay, 2017). People refer to their values when judging what is right and wrong (Roccas et al., 2002), and values are closely related to morality (Sverdlik et al., 2012; Sverdlik & Rechter, 2020; Vauclair et al., 2015). Given this extensive research, it is reasonable to assume that values will be prone to the BTA effect.

Another central characteristic of values that renders them vulnerable to the BTA effect is their inherent desirability. Research consistently found that people tend to rate themselves as higher than others on desirable attributes and lower than others on undesirable attributes (Alicke, 1985; Sedikides et al., 2003, 2005). The desirability of values, however, is complex, being affected by personal as well as societal factors: Individuals differ in their value priorities, hence some values are more/less *personally desired*; and values differ in the extent to which they are encouraged by society, hence some values are more/less *normatively desired in society*. In what follows, we distinguish between these two forces and elaborate on their role in the BTA effect.

### Personally desired values and the BTA effect

While all values are inherently desirable to some extent (Schwartz, 1992), from a young age, people develop quite stable value hierarchies, prioritizing some values over others (Daniel & Benish‐Weisman, 2019). These value priorities lead to interpersonal differences in the extent to which the same value is prioritized by different individuals (Schwartz, 2012). Research found that people tend to perceive their personally important attributes as better and their personally‐unimportant attributes as worse than those of others (Alicke, 1985). People are highly satisfied with their important values and perceive them as closer to their ideal selves than other attributes (e.g., traits, Roccas et al., 2014). Perceiving themselves as possessing ‘better’ personally desired values than those of others is thus likely to be personally‐rewarding, resulting in the BTA effect.

In addition, people strive to fulfil their important values by approaching situations that facilitate their attainment and avoiding situations that hinder it (Hanel et al., 2024). Thus, individuals frequently observe themselves enacting values that are more personally desired and only infrequently observe themselves enacting values that are less personally desired. In contrast, individuals are likely to observe others enacting these values because every value is highly important to at least some people in society and, across individuals, all values may therefore be enacted in one's surrounding (Hanel et al., 2018). Consequently, for values that are personally desired, individuals are more likely to recall value‐expressive instances for the self than for others, whereas for values of lower personal desirability, they are likely to recall such instances more for others than for the self. This asymmetry in self‐other information recall provides an additional mechanism underlying the BTA effect (Chambers & Windschitl, 2004).

### Normatively desired values in society and the BTA effect

Similar to personal value priorities, societies also differ in how much they endorse the same value (Fischer & Schwartz, 2011; Schwartz, 2004). There are also notable similarities across societies, indicating that some values (e.g., benevolence) are more normatively‐desired and some (e.g., power) are less normatively‐desired in most societies (Schwartz & Bardi, 2001). Research found that the BTA effect is stronger for attributes that are desired in society (Sedikides & Gregg, 2008). For example, Sedikides et al. (2003) found that Americans perceived themselves as higher than others on individualistic attributes, whereas Japanese people perceived themselves as higher than others on collectivistic attributes. Research that directly examined self‐other comparisons in personal values found that individuals perceived themselves as higher than others on values desired in society (e.g., benevolence, self‐direction) and as lower than others on values that are less desired in society (e.g., power) (Hanel et al., 2018). People are socialized to internalize what is important in their societies (Tsai et al., 2007). Thus, it is likely to be socially rewarding for people to perceive themselves as endorsing normatively desired values in society more, and less desired values in society less, compared with others, thereby amplifying the BTA effect.

In addition, societal value priorities are translated into norms, behavioural standards and practices that regulate everyday life (Schwartz, 2004). Societies encourage norms and behaviours congruent with desirable values and discourage those congruent with undesirable values. Repeated exposure to social standards leads people to accumulate norm‐congruent memories of their own behaviour, increasing the likelihood of such behaviour in the future (Germar & Mojzisch, 2019). Regarding others, people are especially sensitive to and experience strong negative emotions following violations of social standards by their group members (Ohbuchi et al., 2004; Stamkou et al., 2019). People tend to attribute undesirable behaviours (e.g., those that are incompatible with normatively desired values in society or compatible with less normatively desired ones) to group members more than beneficial behaviours (Hertz, 2021). Moreover, there is evidence that individuals pay attention to and remember negative events more than positive ones (Baumeister et al., 2001). Thus, in the self‐other comparisons of values that are desired in society, people are likely to recall more instances of congruent behaviours of themselves than of others, whereas for values that are less desired in society they are likely to recall more instances when others acted on these values than they did themselves. We therefore reason that the more a value is normatively desired in society, the more it is likely to be rated higher for the self than for others; and the less a value is normatively desired in society, the more it is likely to be rated higher for others than for the self.

Following the rationale of personally desired values alone, one should expect that those who attribute high importance to a value would perceive it as more important to themselves than to others; those who attribute low importance to a value would perceive it as more important to others than to themselves; and this tendency would be expressed to the same extent for all values, regardless of their normative desirability in society. In contrast, following the rationale of the normative desirability of values in society alone, one should expect that, overall, individuals would perceive values that are desired in society as more important to themselves than to others; overall, individuals would perceive values that are less desired in society as more important to others than to themselves, regardless of their personal values. We reason, however, that both personal and societal factors are likely to influence the BTA effect in values, having an additive impact. Hence, it is reasonable to hypothesize that:

Individuals who attribute high importance to a value will rate it as more important to the self than to others, whereas individuals who attribute low importance to a value will rate it as more important to others than to the self.

The BTA effect will be more positive (i.e., more positive self‐others' difference) for values that are more normatively desired in society; and it will be more negative (i.e., more negative self‐others difference) for values that are less normatively desired in society.

## STUDIES OVERVIEW

To test the hypotheses, we conducted three studies that vary in their design and population. There is a debate in the literature regarding whether the BTA effect is a solely Western phenomenon (Hamamura et al., 2007; Heine & Hamamura, 2007) or a universal tendency (Sedikides et al., 2003, 2005). To explore the generalizability of the BTA effect in values, we collected five samples from four cultures that vary on individualism–collectivism. Based on previous research (Hofstede, 1991, 2022): The USA was chosen as representing an individualistic culture, China and Malaysia as representing collectivistic cultures, and Israel as representing a culture in the middle of the individualism–collectivism continuum. This allowed us to investigate commonalities and differences in the BTA effect across cultural groups with different value priorities.

There are two main ways of measuring the BTA effect—a direct and an indirect way (Windschitl et al., 2008). In the direct measurement, participants are instructed to compare the focal construct to others on a single scale, indicating how much this construct characterizes them compared with others. In the indirect measurement, participants rate the focal construct twice, once about themselves and once about others, and the comparison is assessed by the researcher. BTA effects are usually stronger with direct than indirect measurements (Zell et al., 2020). Relative to the indirect measurement, the direct measurement involves a direct comparison, likely to facilitate the contrast effect (Mussweiler, 2003). Moreover, the direct measurement is more prone to demand characteristics, because participants may interpret the direct comparison as a signal that the research primarily focuses on self‐other discrepancies (Alicke & Govorun, 2005). Hence, to isolate the BTA effect and to minimize response biases stemming from contrast effect and demand characteristics, we employed the more conservative indirect measurement. Following Heck and Krueger (2015) and similar to previous research (Bouman et al., 2020; Hanel et al., 2018), in all studies, participants rated both their own values and the values of others to determine how important each value is to them and how much they perceive others to hold the same values. To further minimize response biases, we conducted Study 2 using a time‐lagged design, measuring the perception of others' values 10 weeks after the assessment of personal values.

Previous studies have found that the BTA effect is stronger in comparisons to relatively abstract groups than to relatively concrete ones (see Alicke & Govorun, 2005), and that it is likely to result in higher self‐esteem (Zell et al., 2020). To replicate these findings for the BTA effect in values, Study 3, conducted among students, was designed to investigate whether the comparison to a relatively abstract group (i.e., one's university) results in a stronger bias than the comparison to a relatively concrete group (i.e., one's department); and whether engaging in the BTA bias results with higher self‐esteem.

We report all manipulations, measures and exclusions in all studies. The studies were not pre‐registered. Sensitivity analyses are presented in the Supporting Information. For all studies, data and code can be accessed at https://osf.io/489bc/?view_only=ee897d25e6d044349df5d291842bf45b.

## STUDY 1

In Study 1, we aimed to investigate the generalizability of the BTA effect across cultures that vary on individualism–collectivism (Hofstede, 1991, 2022). Specifically, we recruited participants from the USA (individualistic culture) and China and Malaysia (collectivistic cultures), which should differ in their normative value hierarchies (Schwartz, 2004; Schwartz & Bardi, 2001).

### Method

#### Participants and procedure

Adult residents of the USA, Malaysia and China were recruited through a commercial online panel. They provided informed consent and were paid to answer a 20‐min survey, including personal values, the values of most other people, and socio‐demographics. In total, 993 respondents completed the survey: 361 from the USA (*M*_age_ = 45; *SD* = 13; 63% female); 286 from China (*M*_age_ = 39; *SD* = 12; 49% female); and 346 from Malaysia (*M*_age_ = 33; *SD* = 9; 61% female).

#### Measures

*Personal values* were measured using the Schwartz refined portrait values questionnaire (Schwartz et al., 2017). This scale includes 57 gender‐matched items in randomized order, presented as one‐sentence portraits of different people. Respondents were asked to think about how much that person is like them and respond on a 6‐point scale (1 = not like me at all; 6 = very much like me). Adopting the framing used by Heckhausen and Krueger (1993), *others' values* were measured in the same manner, but respondents were asked to report how similar most other people are to the same set of descriptions on a 6‐point scale (1 = not like most others at all; 6 = very much like most others). Cronbach's alphas for the 10 personal values ranged from .67 for self‐direction in Malaysia to .91 for conformity in China; for others' values they ranged from .63 for stimulation in Malaysia to .94 for conformity in the USA (see Tables 1, 2, 3).

**TABLE 1:** Means, standard deviations, and ranks of personal and others' values in Study 1 (USA).

Values	Personal				Others			
	Rank	*M*	*SD*	Cronbach's alpha	Rank	*M*	*SD*	Cronbach's alpha
Benevolence	1	5.06	0.80	.86	6	4.54	0.95	.91
Universalism	4	4.66	0.90	.76	7	4.14	1.10	.80
Self‐direction	2	4.96	0.77	.80	3	4.64	0.98	.81
Stimulation	9	4.08	1.07	.72	6	4.31	1.00	.71
Hedonism	5	4.55	0.98	.90	2	4.71	0.94	.90
Achievement	8	4.23	1.06	.81	4	4.59	0.94	.88
Power	10	2.99	1.27	.87	8	4.07	1.22	.91
Security	3	4.93	0.80	.76	1	4.77	0.89	.87
Conformity	7	4.34	1.06	.90	10	4.04	1.14	.94
Tradition	6	4.37	0.90	.84	9	4.05	1.07	.89

*Note*: *N* = 361.

**TABLE 2:** Means, standard deviations, and ranks of personal and others' values in Study 1 (China).

Value	Personal				Others			
	Rank	*M*	*SD*	Cronbach's alpha	Rank	*M*	*SD*	Cronbach's alpha
Benevolence	3	4.52	0.85	.88	3	4.30	0.83	.91
Universalism	4	4.41	0.83	.75	7	4.07	0.94	.80
Self‐direction	2	4.64	0.80	.65	4	4.23	0.92	.77
Stimulation	9	3.91	1.04	.78	10	3.88	0.99	.73
Hedonism	5	4.34	0.87	.83	2	4.38	0.90	.84
Achievement	7	4.07	1.04	.86	5	4.22	0.92	.87
Power	10	3.45	1.03	.84	8	4.03	0.94	.88
Security	1	4.76	0.83	.81	1	4.75	0.86	.84
Conformity	6	4.34	0.84	.91	6	4.08	0.93	.93
Tradition	8	4.03	0.86	.87	9	3.94	0.88	.87

*Note*: *N* = 286.

**TABLE 3:** Means, standard deviations, and ranks of personal and others' values in Study 1 (Malaysia).

Value	Personal				Others			
	Rank	*M*	*SD*	Cronbach's alpha	Rank	*M*	*SD*	Cronbach's alpha
Benevolence	2	4.81	0.75	.80	3	4.80	0.78	.87
Universalism	6	4.52	0.74	.73	8	4.53	0.88	.70
Self‐direction	5	4.52	0.77	.67	4	4.66	0.78	.66
Stimulation	8	4.39	0.87	.68	7	4.53	0.84	.63
Hedonism	9	4.21	0.95	.81	5	4.59	0.85	.83
Achievement	4	4.58	0.80	.83	2	4.81	0.74	.90
Power	10	3.30	1.03	.81	10	4.19	0.97	.91
Security	1	4.95	0.73	.71	1	5.00	0.80	.84
Conformity	3	4.71	0.77	.84	6	4.53	0.94	.91
Tradition	7	4.45	0.73	.82	9	4.49	0.87	.87

*Note*: *N* = 346.

### Analytic strategy

#### The role of personally desired values

To test the role of personally desired values in the BTA effect (H1), we conducted correlational analyses. First, for each value, we computed a self‐others difference score by subtracting the perceived importance of the value to others from its importance to the self. We then examined correlations between personal values and their corresponding self‐others' difference scores. A positive association would indicate that the more personally important a value is, the more strongly it is rated as more important for the self than for others, whereas values of lower personal importance would be rated as less important for the self than for others. This approach, however, may be subject to statistical dependency because personal value ratings are included in both the predictor and difference score.

To overcome this dependency and simplify interpretation of the results, we conducted a complementary analysis, following the procedure applied by Smither et al. (1995) in a similar design. For each value, we trichotomized participants into three groups based on their relative importance attributed to a value—low‐, middle‐ and high‐priority groups. We then conducted a GLM analysis for each value with these three groups as a between‐subject factor and ratings of personal and others' values as a within‐subject factor. Based on pairwise comparisons within each group, we expect that in the high‐priority group, personal values would be rated higher than others' values, whereas in the low‐priority group, others' values would be rated higher than personal values. This analysis will also allow us to identify systematic response biases. If responses are biased, participants in all groups would rate themselves as higher/lower than others; and if the pattern of responses in the high‐ and low‐priority groups is aligned with our theoretical reasoning, then it would support the BTA explanation.

#### The role of normatively desired values

To test the hypothesis that the magnitude of the BTA effect depends on the extent to which a value is desired in society (H2), we conducted correlation analyses. To operationalize the magnitude of the BTA effect, we used effect sizes (Cohen's *d*) from the pairwise comparisons within each of the value‐priority groups from the complementary GLM analysis (30 effect sizes for each sample). Positive effect sizes indicate that the self is rated higher than others, and negative effect sizes indicate that others are rated higher than the self, and the absolute value of effect sizes indicates the strength of the BTA effect.

Following a common practice in cross‐cultural research (Hofstede, 1991; Schwartz, 2004), to operationalise the desirability of values in society, we aggregated (averaged) *personal* value scores of the participants in each sample (see Tables 1, 2, 3, 8, and 10). These aggregated values were coded to reflect their hierarchy, indicating the extent to which values are more/less normatively desired in each society. We further used this hierarchy to investigate the role of normatively desired values in the BTA effect. As an exploratory analysis, we also coded the hierarchy of values based on their importance attributed to others and the universal value hierarchy discovered by Schwartz and Bardi (2001), which was observed in most samples in most cultures (benevolence—1, universalism and self‐direction—2.5, security—4, conformity—5, achievement—6, hedonism—7, stimulation—8, tradition—9, power—10). In the analyses, value hierarchies were reversed, so higher scores indicate higher value priority. We expect a positive correlation between BTA effect sizes and aggregated value rankings: The more a value is normatively desired in society, the stronger and more positive the BTA effect would be; and the less a value is normatively desired in society, the stronger and more negative the BTA effect would be.

#### Raw versus ipsatized value scores

The use of rating scales to measure values is susceptible to individual differences in response style (Schwartz, 2016). To address this response‐style bias, scholars have recommended ipsatizing value scores by subtracting each individual's mean value rating from each specific value score prior to analysis (Sagiv & Schwartz, 2022; Schwartz, 2012, 2016). Ipsatization, however, has been criticized as it introduces dependency among value scores and changes their interpretation, shifting the meaning from absolute to relative importance within the individual's hierarchy (Fischer, 2004; Rudnev, 2021). To focus on the absolute importance of values and allow a conceptual comparison of our findings to other BTA studies that typically used raw scores, we report the results for the raw value scores. At the same time, to be compatible with theoreticians' understanding of values as existing in a system in which the crucial element of a value is its relative importance in the value system, we complement these with the analyses of the ipsatized scores, reported in the Supporting Information. Overall, the results were highly consistent across the two strategies, highlighting the robustness of the BTA effect in personal values.

### Results and discussion

Tables 1, 2, 3 present descriptive statistics for the ratings of the 10 personal and others' values. Power was the least important value in all samples, whereas benevolence was the most important in the USA (individualistic) sample and security was the most important in the Chinese and Malaysian (collectivistic) samples. The standard deviations of personal values and others' values were similar. However, participants differentiated somewhat better among their personal value priorities than among the value priorities of others (see Supporting Information). In all samples, personal values and their corresponding others' values were positively correlated (USA $\bar{r}$(358) = .45^1^; China $\bar{r}$(284) = .61; Malaysia $\bar{r}$(344) = .49) (for all correlations, see Tables S1–S3).

#### Personally desired values and the BTA effect

The results of the correlational analyses were highly consistent across three samples, supporting our hypothesis (H1). In all samples, personal values were strongly and positively correlated (all *p*s < .01) with their corresponding self‐others' differences (USA $\bar{r}$(358) = .48; China $\bar{r}$(284) = .43; Malaysia $\bar{r}$(344) = .48; see Table 4). Specifically, the correlations ranged from *r* = .36, *p* < .001 for tradition to *r* = .62, *p* < .001 for achievement in the USA; from *r* = .33, *p* < .001 for universalism to *r* = .54, *p* < .001 for achievement in China; and from *r* = .36, *p* < .001 for conformity to *r* = .57, *p* < .001 for hedonism and achievement in Malaysia (for all correlations, see Tables S4–S6). These results indicate that the more personally important a value was, the more it was perceived as higher for the self than for others, and the less personally important a value was, the more it was perceived as lower for the self than for others.

**TABLE 4:** Correlations between personal values and their corresponding self‐others' difference in Studies 1–3.

Values	Self‐others' differences				
	Study 1 (USA)	Study 1 (China)	Study 1 (Malaysia)	Study 2 (USA)	Study 3 (Israel)
Benevolence	.41**	.44**	.46**	.61**	.44**
Universalism	.45**	.33**	.38**	.61**	.45**
Self‐direction	.36**	.41**	.48**	.57**	.48**
Stimulation	.55**	.49**	.55**	.78**	.78**
Hedonism	.56**	.44**	.57**	.68**	.64**
Achievement	.62**	.54**	.57**	.72**	.58**
Power	.59**	.52**	.55**	.69**	.62**
Security	.39**	.35**	.43**	.65**	.57**
Conformity	.45**	.38**	.36**	.68**	.53**
Tradition	.36**	.40**	.37**	.74**	.65**
$\bar{r}$	.48	.43	.48	.68	.58

*Note*: Study 1: USA *n* = 361, China *n* = 286, Malaysia *n* = 346; Study 2: USA *n* = 363; Study 3: Israel *n* = 227.

** *p* < .01.

The complementary GLM analyses with three value‐priority groups as a between‐subjects factor, and personal and others' values as a within‐subjects factor generally supported our hypothesis as well. For all values in all samples, the interaction was highly significant, indicating that the difference between personal and others' values depended on the personal value priority (USA: *F*s(2, 358) ≥ 17.08, *p*s < .001, *η*s^2^_partial_ ≥ .087; China: *F*s(2, 283) ≥ 11.38, *p*s < .001, *η*s^2^_partial_ ≥ .074; Malaysia: *F*s(2, 343) ≥ 17.77, *p*s < .001, *η*s^2^_partial_ ≥ .094). To decompose these interaction effects, for each value, we conducted a pairwise comparison between the self and others' values within each priority group (see Tables 5, 6, 7). Supporting our hypothesis, in the high‐priority group, most values (27/30 effects) were perceived as more important to the self than to others (average effect size in the USA $\bar{d}$ = 0.54; China $\bar{d}$ = 0.54; Malaysia $\bar{d}$ = 0.33), whereas in the low‐priority group, most values (23/30 effects) were perceived as more important to others than to the self (average effect size in the USA $\bar{d}$ = −0.48; China $\bar{d}$ = −0.42; Malaysia $\bar{d}$ = −0.73).

**TABLE 5:** Pairwise comparisons between personal and others' values in the three value priority groups (high, middle, low) in Study 1 (USA).

Value	Sample rank	Low‐priority group				Middle‐priority group				High‐priority group				Interaction effect, *F*(2, 358)	*η* ^2^_partial_
		Mean diff.	*SE*	*n*	*d*	Mean diff.	*SE*	*n*	*d*	Mean diff.	*SE*	*n*	*d*		
Benevolence	1	0.10	0.08	107	0.12	0.63***	0.08	140	0.67	0.75***	0.08	114	0.83	17.08***	.087
Universalism	4	−0.04	0.08	119	−0.04	0.59***	0.08	117	0.64	0.98***	0.12	125	0.72	27.17***	.132
Self‐direction	2	−0.07	0.08	119	−0.08	0.38***	0.07	136	0.46	0.68***	0.10	106	0.66	20.31***	.102
Stimulation	9	−0.97***	0.10	104	−0.95	−0.20**	0.08	130	−0.23	0.36***	0.07	127	0.45	63.30***	.261
Hedonism	5	−0.77***	0.10	115	−0.70	−0.08	0.06	151	−0.10	0.44***	0.09	95	0.53	46.39***	.206
Achievement	8	−1.15***	0.10	124	−1.06	−0.15*	0.07	134	−0.19	0.36***	0.08	103	0.45	83.09***	.317
Power	10	−2.07***	0.13	118	−1.42	−1.12***	0.10	119	−1.03	−0.11	0.07	124	−0.14	91.89***	.339
Security	3	−0.25**	0.08	109	−0.30	0.27***	0.06	144	0.36	0.44***	0.07	108	0.63	24.43***	.120
Conformity	7	−0.19^†^	0.10	119	−0.18	0.34***	0.08	125	0.39	0.79***	0.11	117	0.68	26.18***	.128
Tradition	6	−0.16	0.10	106	−0.16	0.46***	0.07	150	0.54	0.60***	0.10	105	0.60	20.04***	.101

*Note*: *N* = 361. Mean difference is calculated as personal values minus others' values.

^†^ *p* < .10;

* *p* < .05;

** *p* < .01;

*** *p* < .001.

**TABLE 6:** Pairwise comparisons between personal and others' values in the three value priority groups (high, middle, low) in Study 1 (China).

Value	Sample rank	Low‐priority group				Middle‐priority group				High‐priority group				Interaction effect, *F*(2, 283)	*η* ^2^_partial_
		Mean diff.	*SE*	*n*	*d*	Mean diff.	*SE*	*N*	*d*	Mean diff.	*SE*	*n*	*d*		
Benevolence	3	−0.15*	0.07	87	−0.22	0.26***	0.06	118	0.42	0.54***	0.07	81	0.83	24.57***	.148
Universalism	4	0.09	0.07	93	0.13	0.31***	0.07	93	0.45	0.61***	0.09	100	0.67	11.38***	.074
Self‐direction	2	−0.05	0.06	101	−0.09	0.48***	0.07	94	0.73	0.84***	0.11	91	0.79	30.41***	.177
Stimulation	9	−0.43***	0.07	102	−0.57	0.07	0.08	96	0.09	0.52***	0.10	88	0.56	31.04***	.180
Hedonism	5	−0.42***	0.08	112	−0.49	0.07	0.08	86	0.09	0.36***	0.07	88	0.53	25.95***	.155
Achievement	7	−0.71***	0.08	86	−0.94	−0.17*	0.08	95	−0.22	0.32***	0.07	105	0.43	43.68***	.236
Power	10	−1.02***	0.10	93	−1.11	−0.66***	0.07	100	−0.94	−0.05	0.07	93	−0.08	37.20***	.208
Security	1	−0.25***	0.07	91	−0.37	−0.04	0.05	107	−0.07	0.32***	0.07	88	0.47	18.40***	.115
Conformity	6	−0.08	0.07	106	−0.11	0.34***	0.07	87	0.49	0.58***	0.10	93	0.61	17.88***	.112
Tradition	8	−0.24***	0.06	101	−0.41	0.09	0.07	84	0.13	0.41***	0.07	101	0.56	23.74***	.144

*Note*: *N* = 286. Mean difference is calculated as personal values minus others' values.

* *p* < .05;

*** *p* < .001.

**TABLE 7:** Pairwise comparisons between personal and others' values in the three value priority groups (high, middle, low) in Study 1 (Malaysia).

Value	Sample rank	Low‐priority group				Middle‐priority group				High‐priority group				Interaction effect, *F*(2, 343)	*η* ^2^_partial_
		Mean diff.	*SE*	*n*	*d*	Mean diff.	*SE*	*n*	*d*	Mean diff.	*SE*	*n*	*d*		
Benevolence	2	−0.34***	0.07	120	−0.46	0.02	0.06	119	0.03	0.39***	0.07	107	0.55	32.18***	.158
Universalism	6	−0.34***	0.07	111	−0.47	−0.06	0.07	107	−0.08	0.32***	0.08	128	0.37	21.25***	.110
Self‐direction	5	−0.54***	0.07	117	−0.72	−0.15*	0.06	106	−0.24	0.25***	0.07	123	0.34	37.74***	.180
Stimulation	8	−0.70***	0.07	130	−0.86	0.04	0.08	101	0.05	0.35***	0.07	115	0.46	58.23***	.253
Hedonism	9	−0.99***	0.08	125	−1.08	−0.20**	0.07	118	−0.27	0.16**	0.05	103	0.29	68.66***	.286
Achievement	4	−0.76***	0.08	109	−0.94	−0.18**	0.07	89	−0.28	0.14*	0.05	148	0.21	51.08***	.229
Power	10	−1.54***	0.10	114	−1.44	−0.95***	0.07	110	−1.22	−0.23***	0.07	122	−0.31	65.41***	.276
Security	1	−0.40***	0.06	127	−0.57	−0.04	0.06	112	−0.07	0.36***	0.07	107	0.47	35.13***	.170
Conformity	3	−0.18*	0.08	113	−0.20	0.21***	0.06	126	0.30	0.50***	0.09	107	0.53	17.77***	.094
Tradition	7	−0.40***	0.07	107	−0.52	−0.02	0.06	136	−0.03	0.31***	0.08	103	0.40	23.24***	.119

*Note*: *N* = 346. Mean difference is calculated as personal values minus others' values.

* *p* < .05;

** *p* < .01;

*** *p* < .001.

There was a notable exception. In the high‐priority group, power was perceived as equally important to the self and others in the USA and China, and even as more important to others than to the self in Malaysia. In the middle‐priority group, the results were mixed: some of the values were perceived as more important to the self, some as more important to others, and some as equally important to the self and others.

Taken together, these results support the hypothesis regarding the role of personally‐desired values in the BTA effect: Individuals who attribute high importance to a value perceive it as more important to the self than to others, whereas individuals who attribute low importance to a value perceive it as more important to others than to the self. However, the results for power, which was the least important value in all samples, demonstrate that even those who attribute high importance to this value perceive it as equally or more important to others, providing some initial evidence that the normative desirability of a value in society may amplify the BTA effect.

#### Normatively desired values in society and the BTA effect

As hypothesized (H2), in all samples, the strength of the BTA effect was positively correlated with value desirability in society (USA: *r*(28) = .56, *p* = .001; China: *r*(28) = .41, *p* = .024; Malaysia: *r*(28) = .38, *p* = .039). Interestingly, the results were very similar when correlated with the universal value hierarchy (USA: *r*(28) = .51, *p* = .004; China: *r*(28) = .44, *p* = .015; Malaysia: *r*(28) = .31, *p* = .093) and, as expected, far from significant when correlated with the hierarchy of values based on the perception of others (USA: *r*(28) = .01, *p* = .969; China: *r*(28) = .14, *p* = .450; Malaysia: *r*(28) = .20, *p* = .302).

Taken together, the results supported the hypotheses, indicating that the extent to which a value is personally desired and normatively desired have a joint effect on the strength and direction of the BTA effect. Specifically, the more personally important a value was, the more it was perceived as more important to the self than to others, and this effect was stronger for values that are normatively desired in society. In contrast, the less personally important a value was, the more it was perceived as more important to others than to the self, and this effect was stronger for values that are less normatively desired in society. Interestingly, for the least normatively desired value (i.e., power), the effect of its desirability in society surpassed that of personal importance, as even those who attributed high importance to this value perceived it as more (or equally) important to others than to the self. Overall, the results were virtually identical in all samples and with the sample‐specific and universal value hierarchies, pointing to the universality of the BTA effect in values across cultures.

## STUDY 2

In Study 1, personal and others' values were measured concurrently, a design that may suffer from response‐style biases, such as contrast or assimilation effects (Mussweiler, 2003). To eliminate this alternative explanation, in Study 2, we aimed to replicate our results in a time‐lagged design, in which personal values were assessed 10 weeks prior to the assessment of others' values.

### Method

#### Participants and procedure

Study 2 was conducted in the USA, and its procedure and focal measures were identical to Study 1 with one key difference: Personal and others' values were assessed in different sessions with a 10‐week time lag between them (for more details, see Supporting Information). In total, 363 MTurkers participated in both sessions (*M*_age_ = 35; *SD* = 11; 52% female; 97% born in the USA and 92% having either a mother (89%) or father (91%) born in the USA).

### Results and discussion

Table 8 presents descriptive statistics for the ratings of the 10 personal and others' values. Correlations between personal values and their corresponding others' values were somewhat lower than in Study 1 ($\bar{r}$(361) = .29), ranging from *r* = .17, *p* < .010 for stimulation to *r* = .47, *p* < .001 for security (for all correlations, see Table S7). These results were probably due to the time‐lagged design, which is likely to reduce the potential assimilation effect. To test the hypotheses, we conducted the same analyses as in Study 1.

**TABLE 8:** Means, standard deviations, and ranks of personal and others' values in Study 2 (USA).

Value	Personal				Others			
	Rank	*M*	*SD*	Cronbach's alpha	Rank	*M*	*SD*	Cronbach's alpha
Benevolence	2	4.90	0.80	.87	4	4.49	0.73	.87
Universalism	3	4.41	0.84	.84	10	3.45	0.84	.75
Self‐direction	1	4.96	0.73	.82	5	4.30	0.82	.81
Stimulation	8	3.81	1.17	.71	7	3.83	0.84	.68
Hedonism	5	4.35	0.98	.88	1	4.59	0.84	.88
Achievement	6	4.06	1.00	.82	3	4.56	0.79	.80
Power	10	2.75	1.08	.87	6	3.97	0.93	.88
Security	4	4.40	0.89	.75	2	4.57	0.73	.74
Conformity	7	3.95	1.00	.87	8	3.63	0.86	.91
Tradition	9	3.70	0.94	.88	9	3.61	0.72	.83

*Note*: *N* = 363.

#### Personally desired values and the BTA effect

The results were highly consistent, fully supporting the hypothesis and replicating the findings from Study 1. As hypothesized (H1), personal values were strongly and positively correlated with their corresponding self‐others' differences ($\bar{r}$(361) = .68, see Table 4), ranging from *r* = .57, *p* < .001 for self‐direction to *r* = .78, *p* < .001 for stimulation (for all correlations, see Table S8). These correlations were somewhat stronger than in Study 1, probably indicating that reducing potential response biases in a time‐lagged design can strengthen the BTA effect.

The complementary GLM analyses generally supported our hypothesis as well. For all values, the interaction was highly significant, indicating that the difference between personal and others' values depended on the personal value priority (*F*s(2, 360) ≥ 62.28, *p*s < .001, *η*s^2^_partial_ ≥ .257). As hypothesized, in the high‐priority group, most values (9/10 effects) were perceived as more important to the self than to others (average effect size $\bar{d}$ = 0.91), whereas in the low‐priority group, most values (7/10 effects) were perceived as more important to others than to the self (average effect size $\bar{d}$ = −0.83) (Table 9). Similar to Study 1, power values were perceived as equally important to others, even in the high‐priority group, and universalism values were perceived as more important to the self, even in the low‐priority group, an effect that we did not observe in the USA sample in Study 1. Once again, in the middle‐priority group, the results were inconsistent.

**TABLE 9:** Pairwise comparisons between personal and others' values in three value priority groups (high, middle, low) in Study 2 (USA).

Value	Sample rank	Low‐priority group				Middle‐priority group				High‐priority group				Interaction effect, *F*(2, 360)	*η* ^2^_partial_
		Mean diff.	*SE*	*n*	*d*	Mean diff.	*SE*	*n*	*d*	Mean diff.	*SE*	*n*	*d*		
Benevolence	2	−0.09	0.07	137	−0.11	0.45***	0.06	93	0.80	0.90***	0.07	133	1.19	62.28***	.257
Universalism	3	0.29***	0.08	115	0.35	0.93***	0.07	132	1.21	1.64***	0.09	116	1.65	69.65***	.279
Self‐direction	1	0.00	0.06	124	0.01	0.75***	0.08	110	0.85	1.21***	0.08	129	1.32	66.25***	.269
Stimulation	8	−1.25***	0.08	111	−1.41	0.01	0.07	145	0.01	1.22***	0.10	107	1.23	204.42***	.532
Hedonism	5	−1.12***	0.11	110	−1.01	−0.22***	0.06	130	−0.32	0.52***	0.07	123	0.68	104.90***	.368
Achievement	6	−1.46***	0.10	106	−1.45	−0.53***	0.06	130	−0.72	0.32***	0.07	127	0.42	133.66***	.426
Power	10	−2.09***	0.10	113	−1.90	−1.43***	0.08	137	−1.53	−0.10	0.07	113	−0.12	242.39***	.574
Security	4	−0.85***	0.08	110	−1.08	−0.13*	0.05	141	−0.21	0.46***	0.06	112	0.69	103.04***	.364
Conformity	7	−0.50***	0.08	127	−0.55	0.38***	0.08	107	0.48	0.98***	0.09	129	1.12	99.67***	.356
Tradition	9	−0.80***	0.07	115	−1.12	0.11^†^	0.06	121	0.17	0.88***	0.09	127	0.92	137.70***	.433

*Note*: *N* = 363. Mean difference is calculated as personal values minus others' values.

^†^ *p* < .10;

* *p* < .05;

*** *p* < .001.

#### Normatively desired values in society and the BTA effect

As hypothesized (H2), and replicating the results in Study 1, the strength of the BTA effect (operationalized as Cohen's *d*s) was positively correlated with the sample's value hierarchy (*r*(28) = .47, *p* = .009) and with the universal value hierarchy (*r*(28) = .49, *p* = .006), but not with the hierarchy of values based on the perception of others (*r*(28) = −.24, *p* = .193).

Overall, the results fully replicated the hypotheses and were even stronger when personal and others' values were measured at different time points with a 10‐week period in between. This indicates that the findings from Study 1 were unlikely to be due to contrast or assimilation biases.

## STUDY 3

Study 3 was conducted in a different country, Israel, which has a medium score on the individualism–collectivism continuum (Hofstede, 1991, 2022). In this study, we additionally aimed at testing two phenomena of the BTA effect found in previous research.

Stronger BTA effects were found in comparisons to relatively abstract groups than to relatively concrete groups (Alicke & Govorun, 2005). This is because the BTA effect occurs more with less familiar individuals, where perceivers may rely more on ‘rules of thumb’ than on actual interactions with other group members. In our study, we examined two reference groups that varied in familiarity—fellow students in one's department, and the more distant group of students in the whole university. Thus, it is reasonable to hypothesize that (*H3*) *the BTA effect will be stronger in comparisons to students from the university than to students from one's department*.

Another phenomenon found previously is the association between the BTA effect and self‐esteem (Zell et al., 2020). The theoretical explanation for this link is that BTA helps people feel better about themselves (Alicke et al., 1995). Given the rationale that self‐esteem is a product of BTA, it should be enhanced after an opportunity to manifest the BTA effect. Consequently, it is reasonable to hypothesize that (*H4*) *self‐esteem will be higher for participants who rated values before completing the self‐esteem measure than for participants who completed it before the value rating*.

In addition, in Study 3 we used a different questionnaire (i.e., SVS; Schwartz, 1992) and employed a different wording when assessing others' values, referring to ‘a typical student in one's university/department’. This enabled testing the robustness of the BTA effect beyond a specific value measurement or a particular framing of the reference target.

### Method

#### Participants and procedure

Participants were undergraduate and graduate students from a public university in Israel. They provided informed consent and completed a 20‐min survey including personal values, other students' values, self‐esteem and socio‐demographics. Participation was voluntary. Participants were randomly assigned to a two (a reference group: department/university) by two (self‐esteem scale order: measured before/after the value questionnaires) experimental design. Half of the participants reported self‐esteem before the values questionnaires, and the other half reported it after the values questionnaires. Out of 231 students who agreed to participate, four did not complete the others' values section and were thus excluded from analyses, resulting in a sample of 227 participants (*M*_age_ = 26, *SD* = 4; 57% female). In addition, one participant did not complete the self‐esteem scale.

#### Measures

##### Values

Values were measured with a 44‐item version of the Schwartz Value Survey (SVS), which included all the items that were found to be cross‐culturally equivalent (Schwartz, 1992). Participants rated the importance of each item on a 9‐point scale ranging from −1 (opposed to my values) through 0 (not important) to 7 (of supreme importance). To assess *personal values*, participants indicated the extent to which each item serves as a guiding principle in their life. To assess *others' values*, they indicated the extent to which each item serves as a guiding principle in the life of a typical student in their university/department. The internal reliability of some values (Table 10) was relatively low, albeit mostly within the acceptable range for personal values (Arieli et al., 2016; Davidov et al., 2008).

**TABLE 10:** Means, standard deviations, and ranks of personal and others' values in Study 3 (Israel).

Values	Personal				Others			
	Rank	*M*	*SD*	Cronbach's alpha	Rank	*M*	*SD*	Cronbach's alpha
Benevolence	2	5.11	0.90	.73	8	4.09	1.01	.77
Universalism	6	4.67	0.89	.73	6	4.13	0.99	.81
Self‐direction	1	5.20	0.87	.64	2	4.73	0.93	.70
Stimulation	8	4.28	1.34	.78	7	4.12	1.15	.70
Hedonism	5	4.45	1.31	.60	3	4.54	1.14	.46
Achievement	4	4.86	0.95	.72	1	4.93	0.93	.74
Power	10	3.36	1.35	.67	5	4.28	1.21	.59
Security	5	4.86	1.09	.74	4	4.36	0.99	.66
Conformity	7	4.53	1.21	.75	9	3.87	1.12	.70
Tradition	9	3.46	1.36	.69	10	3.24	1.12	.73

*Note*: *N* = 227.

##### Reference group

To examine the impact of reference group, about half of the participants (*n* = 114) reported values of students in a relatively abstract reference group (one's university). The other half (*n* = 113) reported values of students in a relatively concrete reference group (one's department).

##### Self‐esteem

Self‐esteem was assessed with the Rosenberg (1965) Self‐Esteem scale. Participants indicated the extent of agreement with 10 items (e.g., ‘I feel I have a number of good qualities’; *α* = .79) on a 7‐point scale (1 = completely disagree; 7 = completely agree). About half of the participants (*n* = 124) completed the self‐esteem scale before the value questionnaires (order: self‐esteem scale, own values, others' values), and the remainder of the participants (*n* = 103) completed it after the value questionnaires (order: own values, others' values, self‐esteem scale).

### Results and discussion

Table 10 presents descriptive statistics for the ratings of the 10 personal and others' values. Personal values and their corresponding others' values were positively correlated ($\bar{r}$(225) = .42), ranging from *r* = .16, *p* < .050 for stimulation to *r* = .57, *p* < .001 for conformity (for all correlations, see Table S9). To test the main hypotheses, we conducted the same analyses as in Studies 1–2.

#### Personally desired values and the BTA effect

The results were highly consistent with Studies 1–2. As hypothesized (H1), personal values were strongly and positively correlated with their corresponding self‐others' differences ($\bar{r}$(225) = .58, see Table 4), ranging from *r* = .44, *p* < .001 for benevolence to *r* = .78, *p* < .001 for tradition (for all correlations, see Table S10).

The complementary GLM analyses generally supported our hypothesis as well. For all values, the interaction was highly significant, indicating that the difference between personal and others' values depended on personal value priority (*F*s(2, 224) ≥ 22.00, *p*s < .001, *η*s^2^_partial_ ≥ .164). As hypothesized, in the high‐priority group, most values (9/10 effects) were perceived as more important to the self than to others (average effect size $\bar{d}$ = 0.92), whereas in the low‐priority group, 5 out of 10 values were perceived as more important to others than to the self (average effect size $\bar{d}$ = −0.52) (Table 11). Once again, power values were perceived as more important to others than to the self, even in the high‐priority group. In addition, benevolence values were perceived as more important to the self than to others even in the low‐priority group. As in Studies 1–2, in the middle‐priority group, the results were mixed.

**TABLE 11:** Pairwise comparisons between personal and others' values in the three value priority groups (high, middle, low) in Study 3 (Israel).

Value	Sample rank	Low‐priority group				Middle‐priority group				High‐priority group				Interaction effect, *F*(2, 224)	*η* ^2^_partial_
		Mean diff.	*SE*	*n*	*d*	Mean diff.	*SE*	*n*	*d*	Mean diff.	*SE*	*n*	*d*		
Benevolence	2	0.46***	0.09	74	0.58	1.18***	0.09	85	1.43	1.44***	0.14	68	1.29	22.00***	.164
Universalism	6	−0.02	0.11	74	−0.02	0.58***	0.10	78	0.64	1.04***	0.10	75	1.23	26.20***	.190
Self‐direction	1	−0.08	0.10	78	−0.09	0.68***	0.10	76	0.77	0.85***	0.10	73	1.01	25.27***	.184
Stimulation	8	−1.37***	0.14	65	−1.24	0.38**	0.12	100	0.31	1.38***	0.18	62	0.98	80.27***	.417
Hedonism	5	−1.26***	0.15	65	−1.04	0.00	0.10	92	0.00	0.91**	0.13	70	0.85	161.11***	.590
Achievement	4	−0.66***	0.09	85	−0.78	−0.12	0.10	74	−0.14	0.71***	0.13	68	0.66	17.57***	.274
Power	10	−1.85***	0.14	80	−1.50	−0.96***	0.12	67	−0.94	0.04	0.14	80	0.03	51.48***	.315
Security	5	−0.19	0.12	74	−0.18	0.68***	0.08	87	0.91	1.03***	0.13	66	0.99	32.35***	.224
Conformity	7	0.02	0.10	78	0.02	0.82***	0.10	80	0.95	1.20***	0.14	69	1.04	28.51***	.203
Tradition	9	−0.79***	0.10	78	−0.92	0.29*	0.13	68	0.26	1.15***	0.12	81	1.09	158.29***	.586

*Note*: *N* = 227. Mean difference is calculated as personal values minus others' values.

* *p* < .05;

** *p* < .01;

*** *p* < .001.

#### Normatively desired values in society and the BTA effect

As hypothesized (H2) and replicating our prior results, the strength of the BTA effect (operationalized as Cohen's *d*s) was positively correlated with the sample's value hierarchy (*r*(28) = .39, *p* = .032) and with the universal value hierarchy (*r*(28) = .55, *p* = .002), but not with the hierarchy of values based on the perception of others (*r*(28) = −.18, *p* = .343).

#### Effect of the reference group on BTA

To examine the role of the reference group, for each participant, we first summed absolute self‐others' differences across the 10 values to create a single aggregated score representing the overall magnitude of the BTA effect (*M* = 10.14, Median = 9.24, *SD* = 4.93). There was one outlier, who scored more than 3 standard deviations above the mean (BTA score = 39.96). The results were highly consistent when this outlier was removed (see Supporting Information). We then tested our hypothesis (H3) by comparing this aggregated score between the two randomly assigned reference groups (university versus department) using a *t*‐test. As hypothesized, the BTA effect was significantly stronger when personal values were compared with a more abstract group, university (*M* = 10.97, *SD* = 5.13) than when they were compared with a more concrete group, department (*M* = 9.31, *SD* = 4.58; *t*(225) = 2.57, *p* = .011, *d* = 0.34). Even though the BTA effect was significantly weaker in comparisons to one's department, it was still significantly different from zero (*t*(112) = 21.60, *p* < .001, *d* = 2.03), indicating that people manifested the BTA effect even when comparing themselves to familiar and relatively close others (see also Brown, 1986).

#### Consequence of the BTA effect for self‐esteem

We further aimed to investigate whether self‐esteem is a consequence of the BTA effect. For that aim, we compared the two conditions of self‐esteem order. The participants who reported their self‐esteem before they completed the value questionnaires did so before they had the opportunity to express BTA. Thus, their self‐esteem could not be affected by BTA. Conversely, those who reported self‐esteem after values had the opportunity to manifest the BTA effect, and their self‐esteem could be affected by BTA, at least to some extent. However, the participants varied in the extent to which they expressed the BTA effect, as reflected in their overall BTA score from the previous analysis. To test our hypothesis (H4), we conducted a regression analysis with self‐esteem entered as the dependent variable. The predictors were self‐esteem order (effect coded: ‘self‐esteem before’ condition coded as −1, and ‘self‐esteem after’ condition coded as 1) and the BTA continuous score (standardized) entered in the first step, and their interaction entered in the second step.

The main effects of the self‐esteem order and the BTA score were not significant (*t*s(222) < 1.28, *p*s > .203). As expected, the interaction was significant (*b* = 0.15, *SE* = 0.053, *β* = .18, *t*(222) = 2.72, *p* = .007, 95% *CI* for *b* [0.04, 0.25]), explaining 3.2% of the variance in self‐esteem (*F*_change_(1, 222) = 7.42, *p* = .007). Simple effects revealed that among participants who manifested the BTA effect to a greater extent, self‐esteem was significantly higher after the manifestation than before it (*b* = 0.21, *SE* = 0.077, *t*(222) = 2.74, *p* = .007). In contrast, among participants who manifested it to a lesser extent, there was no difference between the before and after conditions (*b* = −0.08, *SE* = 0.077, *t*(222) = −1.01, *p* = .315). Thus, our findings are consistent with the hypothesis that manifesting the BTA effect increases self‐esteem.

In summary, we replicated our results in a different cultural context—Israel. We also found a stronger BTA effect when comparing personal values to those of others from relatively abstract groups than from relatively concrete groups. Furthermore, we found that manifesting the BTA effect in values has the potential to boost self‐esteem.

## GENERAL DISCUSSION

This paper provided full support for the hypothesized BTA effect in personal values. Across five samples from four cultures that differ in individualism–collectivism, we consistently showed that the more personally important a value is, the more it is likely to be perceived as higher for the self than for others, and the less personally important a value is, the more it is likely to be perceived as higher for others than for the self. This robust effect, however, is augmented by the extent to which a value is desired in society: The self‐other comparison is amplified towards the self for values that are normatively desired in society, and it is amplified towards others for values that are less normatively desired in society. This bias is especially pronounced in comparisons to others from a relatively abstract group than to others from a relatively concrete group. Finally, expressing the BTA effect has positive consequences for self‐esteem, pointing to a motivational aspect of the bias.

Unlike some previous research (Heine & Hamamura, 2007), we did not observe less BTA bias in collectivistic cultures. Our findings revealed that the BTA effect is expressed to a similar extent by members from individualistic, collectivistic and in‐between cultures. In all samples, individuals perceived others as holding less of their personally important values and more of their personally unimportant values than themselves. Personal values are one of the central aspects of the self; they are closely related to one's ideals; and people are highly satisfied with their values (Roccas et al., 2014). These unique features may amplify the BTA effect in personal values in a way that transcends cultural boundaries and is universally expressed. The magnitude of the bias, however, depends on the normative desirability of the different values in society.

Consistent with previous research on other characteristics (Sedikides et al., 2003, 2005), we also found that the BTA effect can be augmented by the extent to which a value is normatively desired in society. Individuals who attributed high importance to desired values in society perceived these values as much more important to the self than to others, and this gap was much smaller for values that are less desired in society. Similarly, individuals who attribute low importance to values that are less desired in society perceived these values as much more important to others than to the self, and this gap was much smaller for values that are desired in society. In some extreme cases, the desirability of a value in society may even override its personal desirability. For power, which was the least important value in all samples, even individuals who attributed relatively high importance to this value saw themselves as equal or lower on it than others.

This pattern was less consistent for the most desired values in society. For example, for self‐direction, which was the most normatively desired value in Study 2 (the USA) and Study 3 (Israel), participants who attributed low importance to this value perceived it as equally important to the self and others than to the self. Whereas for benevolence, which was the second most normatively desired value in Study 3, and for universalism, which was the third most normatively desired value in Study 2, even individuals who attributed low importance to these values perceived them as more important to the self than to others.

Following previous research on other characteristics (Sedikides et al., 2003, 2005), we theorized that the extent to which values are more or less desired in society is defined by the social value hierarchy within a specific cultural group. While our findings supported this assumption, they also demonstrated similar effects for the universal value hierarchy reported in Schwartz and Bardi (2001). Taken together, these results indicate that self‐enhancement may indeed be a universal human phenomenon (Brown, 2003). Future research is still needed to further understand how people achieve positive self‐evaluation by making social comparisons on the basis of more normatively desired values in society.

Similar to previous findings on other characteristics (Alicke et al., 1995), we found stronger BTA effects in rating the values of a less familiar group. This strengthens the evidence that people engage in the BTA effect more when they do not know the comparison group so well. These results are consistent with evidence that individual perceptions regarding values of close others, such as family members or friends, are less prone to BTA bias (Bernard et al., 2006; Dobewall et al., 2014) than their perceptions regarding values of distant others, such as people in their city (Hanel et al., 2018). Our results indicate that the BTA effect is still present in evaluations of others from a relatively familiar group, such as one's academic department. A recent study that investigated how people perceive values of concrete individuals (e.g., those they have very close relationships with) also revealed a different kind of bias, according to which close others' values were perceived as more similar to the self than they actually are (Ginosar Yaari et al., 2025). Thus, researchers should be cautious in using others' reports on values when they are interested in personal values.

The biased perception of others' values has important societal implications, which have already been the subject of some empirical research. For example, the more people think of others as having less moral values (i.e., lower self‐transcendence and higher self‐enhancement), the more they feel estranged from society and tend to engage less in civic action that contributes to society, such as political voting (Sanderson et al., 2019). Thus, the general BTA effect may have negative consequences for society, and researchers should examine ways to potentially mitigate it. For example, Wolf and Hanel (2024) found socially positive consequences of interventions that are intended to show people that their values are similar to those of others, such as improving attitudes towards outgroups.

Our results also highlight some potential contradictions in the literature. On the one hand, in line with the self‐enhancement literature on other characteristics and previous findings (Zell et al., 2020), we have shown that perceiving one's values as different from others in a BTA‐consistent way can increase self‐esteem. On the other hand, research stemming from the person‐environment fit literature has found that value congruency with one's environment is associated with well‐being (reviewed in Sagiv & Schwartz, 2022), whereas perceived gaps between personal and others' values can lead to estrangement (Bernard et al., 2006). There are two potential explanations that can help resolve this puzzle.

First, the congruency effect may be more beneficial for the self when perceived in relationships with close others, in which the BTA effect is weaker. The more our values are similar to those around us, the easier it is for us to get along with them, supporting our happiness (Sortheix & Lönnqvist, 2014). Conversely, congruence with others from distant groups may be less crucial, leaving room for the BTA effect to drive self‐enhancement tendencies.

The second explanation builds on the distinction between more and less desired values in society. For values that are more normatively desired, manifesting the BTA effect by perceiving one's own values as superior to those of others may be especially rewarding and contribute to higher self‐esteem. In contrast, for less normatively desired values, perceived similarity between the self and others may be more rewarding, as it affirms that one's own attributes are not inferior to those of others. Consistent with this explanation, prior research shows that individuals who prioritize self‐enhancement values (power and achievement), which tend to be less desired in society, report higher life satisfaction when they perceive others to also endorse these values (Wolf et al., 2021). While this explanation could be explored in Study 3, it is more appropriate to investigate it in an independent study with a larger sample, and in contexts where value congruence is particularly meaningful (e.g., the workplace). Employing response surface analysis represents a promising methodological approach for future research, as it can provide nuanced insights into value (in)congruence related to well‐being outcomes.

An alternative explanation for differences in self‐enhancement biases in terms of personally desired values may reflect the assessment of the difference between self and other being based more in reality (see Heck & Krueger, 2015). For example, people who attribute a relatively high importance to a value should perceive the average other to attribute a lower importance to that value, and vice versa, and this may be a reflection of reality rather than bias. However, we do not find this for power values across samples, where people who attribute a relatively high importance to power perceive the average other to attribute a higher importance to that value than they do. Given that this value is the least normatively desired in each culture, it provides further evidence of self‐enhancement bias.

We have replicated the results using raw and ipsatized value scores. While ipsatization is generally recommended by major value theoreticians (Sagiv & Schwartz, 2022; Schwartz, 2012, 2016), it has been criticized for introducing spurious dependency among scores (Fischer, 2004; Rudnev, 2021). Our results indicate that the absolute importance of a value (raw scores) and its importance relative to other values (ipsatized scores) reveal similar BTA effects, leading to nearly identical conclusions regarding biased perceptions of others' values. In principle, researchers could carefully consider their scoring method based on the conceptual focus of their study and its design (Borg & Bardi, 2016).

### Limitations and future directions

Our findings support the hypothesized relations across the full spectrum of values and beyond Western cultures. Nonetheless, the use of panel samples may limit the generalizability of our results to people with internet access, who are likely more prevalent in urban than rural locations. Consequently, sample‐specific value hierarchies used as a proxy for the broader socio‐cultural environment may be less representative of the participants' actual environment. Particularly striking was the Chinese value hierarchy, where we did not observe the expected collectivistic structure that would have placed conformity higher in the hierarchy and self‐direction lower in the hierarchy. Although China is typically categorized as a collectivistic culture (Hofstede, 2022), the individualism–collectivism cultural dimension was not explicitly measured in the current research. Therefore, we could not empirically verify the extent to which this dimension influenced the observed value hierarchies or the magnitude of the BTA effect. Even though results were consistent across samples and replicated with the universal value hierarchy, future research can further investigate BTA effects in values using more representative samples and explicit measures of cultural orientations across a wider range of societies.

Another potential limitation is our framing of the reference group in Studies 1–2. Following previous research (Heckhausen & Krueger, 1993), the participants were asked to report on the values of ‘most other people’. Specifically, they were instructed to think about ‘how similar MOST OTHER PEOPLE are to the person in these descriptions … so this question is not about you … rather it is about the average person in general.’ This wording may lead to some ambiguity in reference groups. While some participants could refer to most others from close or familiar groups, for which the BTA effect is usually weaker, others could refer to people from more distant groups, for which the BTA effect is usually stronger. This ambiguity may limit generalization of the observed strength of the effects. To overcome this potential limitation in Study 3, we used specific reference groups and slightly different instructions, focusing on a typical member from well‐defined groups. The findings fully replicated those of Studies 1–2. Future research should investigate the role of reference groups on the magnitude of BTA effects in values.

To reduce demand characteristics and contrast effects (Alicke & Govorun, 2005), we measured the BTA effect indirectly by asking participants to rate values of themselves and others separately. This measure is more conservative and tends to result in weaker BTA effects than direct comparisons (Alicke & Govorun, 2005; Zell et al., 2020). As a result, our findings may be weaker than in real‐life situations, in which people directly compare themselves to others. Future research can investigate how the direct comparison process impacts the magnitude of the BTA effect in values.

### Conclusions

Although people tend to experience greater well‐being when their values are congruent with those of their environment, our findings indicate that they nevertheless perceive themselves as having ‘better’ values than the average person, especially for values that are normatively desired in society, and that this perception is associated with elevated self‐esteem. This pattern highlights the interplay between personal and societal value‐priorities in social comparison processes and demonstrates the complex consequences of these dynamics for the self.

## AUTHOR CONTRIBUTIONS

**Andrey Elster:** Conceptualization; investigation; resources; writing – review and editing; writing – original draft; methodology; visualization; formal analysis; data curation. **Anat Bardi:** Conceptualization; methodology; writing – review and editing; writing – original draft. **Joanne Sneddon:** Conceptualization; writing – original draft; writing – review and editing. **Lilach Sagiv:** Supervision; writing – review and editing; conceptualization. **Sonia Roccas:** Supervision. **Julie Anne Lee:** Conceptualization; formal analysis; funding acquisition; investigation; methodology; project administration; resources; visualization; writing – review and editing; writing – original draft.

## CONFLICT OF INTEREST STATEMENT

The authors declare no conflicts of interest.

## Supporting information


Figure S1.

## Data Availability

For all studies, the materials, data, outputs of the main analyses, and code can be accessed at https://osf.io/489bc/?view_only=ee897d25e6d044349df5d291842bf45b.

## Footnotes

^1^
In all studies, when presenting average correlations, initial correlation coefficients were first transformed using Fisher's *Z* transformation, then averaged and transformed back.
