# Supplementary material for: On being better than average in values
Source: Br J Soc Psychol. 2026 Apr 28;65:e70089. doi: 10.1111/bjso.70089 (PMC13125740; doi:10.1111/bjso.70089)
Supplement: Supplementary file 1 — Figure S1. [file BJSO-65-0-s001.docx]

**On Being Better Than Average in Values**

**SUPPLEMENTAL MATERIAL**

**Schwartz Value Theory (1992)**

According to the Schwartz Value Theory (1992), the circular structure formed by the ten values can be further divided into two bipolar dimensions, with each pole serving as a higher-order value. Specifically, the higher-order value of self-transcendence expresses the motivation to promote and protect others (benevolence and universalism). This higher-order value conflicts with the higher-order value of self-enhancement, which expresses the motivation to enhance oneself even at the expense of others (power, achievement, and sometimes hedonism). The other dimension includes the higher-order value of openness to change, which expresses the motivation for openness to new experiences and thoughts (self-direction, stimulation, and sometimes hedonism). This higher-order value conflicts with the higher-order value of conservation, which expresses the motivation to maintain the status quo (security, conformity, and tradition).

**Sensitivity Analysis**

Sensitivity analysis was conducted to assess sample size adequacy for (a) correlational analysis and (b) repeated measures ANOVA with three groups, using G*Power (Faul et al., [2007](https://bpspsychub.onlinelibrary.wiley.com/doi/full/10.1111/bjso.12634#bjso12634-bib-0005)). In Studies 1-2, the smallest sample size was for China (*N* = 286). Figure 1S(A) shows that a sample of 286 could detect a small effect (ρ = 0.16) in a two-tailed correlational analysis, considering an alpha level of .05 and 80% power. Figure 1S(B) shows that a sample of 286 could detect a small effect (f = 0.09) with a Critical *F* of 3.03 in a repeated measures ANOVA (within-between interaction) with three experimental groups, two measures, and a correlation between repeated measures of .48 (lowest for this sample), considering an alpha level of .05 and 80% power.

**Figure 1S**

*Studies 1-2 Sensitivity Analysis (A) Two-Tailed Correlational Analysis; (B) Repeated Measures ANOVA*

**A**

**B**

In Study 3, the sample size was 227. Figure 2S(A) shows that a sample of 227 could detect a small effect (ρ = 0.18) in a two-tailed correlational analysis, considering an alpha level of .05 and 80% power. Figure 2S(B) shows that a sample of 227 could detect a small effect (f = 0.13) with a Critical *F* of 3.04 in a repeated measures ANOVA (within-between interaction) with three experimental groups, two measures, and a correlation between repeated measures of .16 (lowest for this sample), considering an alpha level of .05 and 80% power.

**Figure 2S**

*Study 3 Sensitivity Analysis (A) Two-Tailed Correlational Analysis; (B) Repeated Measures ANOVA*

**A**

**B**

**Variability in the Rating of Personal and Others’ Values in all Samples**

In each sample, standard deviations of personal values and others’ values were almost identical for all value types (see Tables 1-3, for Study 1; Table 8, for Study 2; and Table 10, for Study 3). These results indicate that for each value type, the distribution of personal value scores and the distribution of perceptions of others’ values were relatively similar. For example, participants differed in their own importance attributed to benevolence similarly to how they differed in their perceptions of benevolence of others. In other words, individuals differed from one another in their perceptions of others to the same extent that they differed in their personal value priorities. The question still remains whether individuals are able to differentiate between the ten value types of others to the same extent that they differentiate between their own ten value types, or whether the differentiation between others’ values is lower/higher than between their own values.

To investigate this question, for each participant, we computed a standard deviation of their ten personal values and a standard deviation of the ten values they attributed to others, indicating the differentiation between personal and others’ values within the individual. We then conducted a pairwise comparison of these differentiation scores within each sample. The differentiation of personal values was somewhat higher than the differentiation of others’ values, demonstrating low-medium effect sizes (Study 1 USA: *M*_personal_ = 0.85, *M*_others_ = 0.65; *t*(360) = 10.41, *p* < .001, *d* = 0.55; China: *M*_personal_ = 0.65, *M*_others_ = 0.54; *t*(285) = 6.91, *p* < .001, *d* = 0.41; Malaysia: *M*_personal_ = 0.64, *M*_others_ = 0.50; *t*(345) = 8.98, *p* < .001, *d* = 0.48; Study 2 USA: *M*_personal_ = 0.99, *M*_others_ = 0.76; *t*(362) = 13.18, *p* < .001, *d* = 0.69; Study 3 Israel: *M*_personal_ = 1.06, *M*_others_ = 0.90; *t*(226) = 5.98, *p* < .001, *d* = 0.40). However, the differentiation of others’ values was still substantially different from zero (Study 1 USA: *t*(360) = 28.17, *p* < .001, *d* = 1.48; China: *t*(285) = 29.39, *p* < .001, *d* = 1.74; Malaysia: *t*(345) = 28.48, *p* < .001, *d* = 1.53; Study 2 USA: *t*(362) = 46.66, *p* < .001, *d* = 2.45; Study 3 Israel: *t*(226) = 37.76, *p* < .001, *d* = 2.51). These results indicate that participants differentiated somewhat better between their personal value priorities than between the value priorities of others.

**Study 1**

**Results for the Ipsatized Value Scores**

The results for the ipsatized value scores were highly consistent across the analyses, fully replicating the results for the raw value scores and supporting our hypotheses. Tables 11S-13S present descriptive statistics for the ratings of the ten ipsatized personal and others’ values. In all samples, personal values and their corresponding others’ values were only moderately correlated (USA $\bar{r}$(358) = .22; China $\bar{r}$(284) = .32; Malaysia $\bar{r}$(344) = .25) (for all correlations, see Tables 14S-16S).

***Personally-Desired Values and the BTA Effect***

As hypothesized (*H1*), in all samples, ipsatized personal values were strongly and positively correlated with their corresponding self-others’ differences (USA $\bar{r}$(358) = .64; China $\bar{r}$(284) = .61; Malaysia $\bar{r}$(344) = .61), ranging from *r* = .50, *p* < .001 for security to *r* = .72, *p* < .001 for stimulation in the USA; from *r* = .31, *p* < .001 for security to *r* = .70, *p* < .001 for benevolence and stimulation in China; and from *r* = .49, *p* < .001 for security to *r* = .72, *p* < .001 for stimulation in Malaysia (for all correlations, see Tables 17S-19S).

The complementary GLM analyses with three value-priority groups as a between-subjects factor, and ipsatized personal and others’ values as a within-subjects factor supported the hypothesis as well. For all values in all samples, the interaction was highly significant, indicating that the difference between personal and others’ values depended on the personal value priority (USA: *F*s(2, 358) ≥ 46.98, *p*s < .001, ηs^2^_partial_ ≥ .208; China: *F*s(2, 283) ≥ 6.40, *p*s < .010, ηs^2^_partial_ ≥ .043; Malaysia: *F*s(2, 343) ≥ 36.45, *p*s < .001, ηs^2^_partial_ ≥ .175). To decompose these interaction effects, for each value, we conducted a pairwise comparison between the self and others’ values within each priority group (see Tables 20S-22S). As hypothesized, in the high-priority group, most values (26/30 effects) were perceived as more important to the self than to others (average effect size in the USA $\bar{d}$ = 0.71; China $\bar{d}$ = 0.67; Malaysia $\bar{d}$ = 0.74), whereas in the low-priority group, most values (27/30 effects) were perceived as more important to others than to the self (average effect size in the USA $\bar{d}$ = -0.62; China $\bar{d}$ = -0.64; Malaysia $\bar{d}$ = -0.61).

Once again, the results for power values showed a different pattern, pointing to the role of normative desirability of values in society. Even in the high-priority group, power was perceived as more important to others than to the self in all samples. In the middle-priority group, the results were mixed: Some of the values were perceived as more important to the self, some as more important to others, and some as equally important to the self and others.

***Normatively-Desired Values in Society and the BTA Effect***

Once again, in all samples, the strength of the BTA effect (operationalized as Cohen’s d) was positively correlated with the sample-based ipsatized value desirability in society (USA: *r*(28) = .52, *p* = .003; China: *r*(28) = .37, *p* = .046; Malaysia: *r*(28) = .37, *p* = .046) as well as with the universal value hierarchy (USA: *r*(28) = .49, *p* = .006; China: *r*(28) = .46, *p* = .010; Malaysia: *r*(28) = .32, *p* = .088). As expected, correlations with the hierarchy of values based on the perception of others were far from significant (USA: *r*(28) = -.03, *p* = .869; China: *r*(28) = .10, *p* = .614; Malaysia: *r*(28) = .16, *p* = .394).

**Study 2**

**Method**

***Procedure***

This study included two parts. Part 1 involved a survey that lasted on average 23 minutes, which included personal values, some unrelated scales and behaviors, followed by several socio-demographic variables. Ten weeks later, the same participants were invited, through a custom-built MTurk messaging application, to complete the second survey, which lasted 13 minutes on average. Part 2 included the same values questionnaire to measure others’ values followed by several unrelated scales for a separate study.

**Results for the Ipsatized Value Scores**

Once again, the results for the ipsatized values scores supported our hypotheses and fully replicated the results for the raw value scores. Table 23S presents descriptive statistics for the ratings of the ten ipsatized personal and others’ values. Correlations between ipsatized personal values and their corresponding others’ values were somewhat lower than the correlations of the raw scores ($\bar{r}$(361) = .15), ranging from *r* = -.03, *p* > .100 for self-direction to *r* = .34, *p* < .001 for security (for all correlations, see Table 24S).

***Personally-Desired Values and the BTA Effect***

As hypothesized, ipsatized personal values were strongly and positively correlated with their corresponding self-others’ differences ($\bar{r}$(361) = .74), ranging from *r* = .66, *p* < .001 for power to *r*(361) = .82, *p* < .001 for stimulation (for all correlations, see Table 25S).

The complementary GLM analyses supported our hypothesis as well. For all values, the interaction was highly significant, indicating that the difference between personal and others’ values depended on the personal value priority (*F*s(2, 360) > 98.54, *p*s < .001, ηs^2^_partial_ > .354). In the high priority group, all but power values were perceived as more important to the self than to others (average effect size $\bar{d}$ = 0.86), whereas in the low priority group, all but universalism values were perceived as more important to others than to the self (average effect size $\bar{d}$ = -0.97) (see Table 26S). Power values were perceived as more important to others, even in the high priority group, and universalism values were perceived as more important to the self, even in the low priority group. In the middle priority group, some values were perceived as more important to the self, and some were perceived as more important to others.

***Normatively-Desired Values in Society and the BTA Effect***

These results were fully replicated for the ipsatized values as well. The strength of the BTA effect (operationalized as Cohen’s *d*s) was positively correlated with the sample’s value hierarchy (*r*(28) = .48, *p* = .008) and with the universal value hierarchy (*r*(28) = .53, *p* = .003), but not with the hierarchy of values based on the perception of others, which was somewhat negative (*r*(28) = -.29, *p* = .114).

**Study 3**

**Results for the Raw Values Scores: Omitting the Outlier Observation**

***Effect of the Reference Group on BTA***

The results were highly consistent when the outlier observation was removed. As hypothesized, the BTA effect was significantly stronger when personal values were compared to a more abstract group, university (*M* = 10.71, *SD* = 4.36) than when they were compared to a more concrete group, department (*M* = 9.31, *SD* = 4.58; *t*(224) = 2.36, *p* = .019, *d* = 0.31). Even though the BTA effect was significantly weaker in comparisons to one’s department, it was still significantly different from zero (*t*(112) = 21.60, *p* < .001, *d* = 2.03).

***Consequence of the BTA Effect for Self-Esteem***

Removing the outlier observation identified in the previous analysis slightly weakened the results. The main effects of the self-esteem order and the BTA score were not significant (*t*s(221) < 1.12, *p*s > .265). The expected interaction became marginally significant (*b* = 0.12, *SE* = 0.062, β = .14, *t*(221) = 1.89, *p* = .059, 95% *CI* for *b* [-0.01, 0.24]), explaining 1.6% of the variance in self-esteem (*F*_change_(1, 221) = 3.59, *p* = .059). Simple effects, however, revealed the identical pattern: Among participants who manifested the BTA effect to a greater extent, self-esteem was significantly higher after the manifestation than before it (*b* = 0.18, *SE* = 0.084, *t*(221) = 2.12, *p* = .036). In contrast, among participants who manifested it to a lesser extent there was no difference between the before and after conditions (*b* = -0.06, *SE* = 0.084, *t*(222) = -0.68, *p* = .496).

**Results for the Ipsatized Value Scores**

As in Studies 1-2, the results for the ipsatized values scores fully replicated the results for the raw value scores. Table 27S presents descriptive statistics for the ratings of the ten ipsatized personal and others’ values. Ipsatized personal values and their corresponding others’ values were only moderately correlated ($\bar{r}$(225) = .21), ranging from *r* = -.04, *p* > .100 for stimulation to *r* = .36, *p* < .001 for hedonism and power (for all correlations, see Table 28S).

***Personally-Desired Values and the BTA Effect***

As hypothesized, ipsatized personal values were strongly and positively correlated with their corresponding self-others’ differences ($\bar{r}$(225) = .65), ranging from *r* = .51, *p* < .001 for achievement to *r* = .78, *p* < .001 for tradition (for all correlations, see Table 29S).

The complementary GLM analyses supported our hypothesis as well. For all values, the interaction was highly significant, indicating that the difference between personal and others’ values depended on personal value priority (*F*s(2, 224) > 26.14, *p*s < .001, ηs^2^_partial_ > .189). In the high-priority group, all but power and achievement values were perceived as more important to the self than to others (average effect size $\bar{d}$ = 0.75), whereas in the low-priority group, all but benevolence values were perceived as more important to others than to the self (average effect size $\bar{d}$ = -0.92) (see Table 30S). Once again, power was perceived as more important to others than to the self, even in the high-priority group. In addition, benevolence was perceived as more important to the self than to others even in the low-priority group. As in Studies 1-2, in the middle-priority group, the results were mixed.

***Normatively-Desired Values in Society and the BTA Effect***

Generally supporting our hypothesis, the strength of the BTA effect (operationalized as Cohen’s *d*s) was positively and marginally correlated with the sample’s value hierarchy (*r*(28) = .32, *p* = .081) and with the universal value hierarchy (*r*(28) = .51, *p* = .004), but not with the hierarchy of values based on the perception of others (*r*(28) = -.23, *p* = .213).

***Effect of the Reference Group on BTA***

As in the main manuscript, we first calculated the overall magnitude of the BTA effect by summing absolute self-others’ differences across all ipsatized values (*M* = 8.78, Median = 7.97, *SD* = 4.01). Based on the Stem-and-Leaf plot, there were nine extreme observations (> 17.50). To test the hypothesis, we removed these observations (*n* = 218, *M* = 8.27, Median = 7.85, *SD* = 3.14) and conducted a *t*-test, comparing between the two reference groups. Replicating the results for the raw scores, the BTA effect was significantly stronger when personal values were compared to a more abstract group (university, *M* = 8.77, *SD* = 3.17) than when they were compared to a more concrete group (department, *M* = 7.76, *SD* = 3.04; *t*(216) = 2.39, *p* = .018, *d* = 0.32). Even though the BTA effect was significantly weaker in comparisons to one’s department, it was still significantly different from zero (*t*(107) = 26.43, *p* < .001, *d* = 2.56).

***Consequence of the BTA Effect for Self-Esteem***

To test our hypothesis, we conducted a regression analysis with self-esteem entered as the dependent variable, self-esteem order (effect coded: “self-esteem before” condition coded as -1, and “self-esteem after” condition coded as 1) and the BTA continuous score (standardized) based on the ipsatized scores from the previous analysis entered in the first step, and their interaction entered in the second step. The main effects of the self-esteem order and the BTA score were not significant (*t*s(213) < 0.89, *p*s > .377). Replicating the results for the raw scores, the interaction was significant (*b* = 0.11, *SE* = 0.054, β = .14, *t*(213) = 2.05, *p* = .042, 95% *CI* for *b* [0.00, 0.22]), explaining 1.9% of the variance in self-esteem (*F*_change_(1, 213) = 4.19, *p* = .042). Simple effects revealed that among participants who manifested the BTA effect to a greater extent, self-esteem was significantly higher after the manifestation of BTA than before it (*b* = 0.16, *SE* = 0.077, *t*(213) = 2.05, *p* = .041). In contrast, among participants who manifested BTA to a lesser extent, there was no difference between the before and after conditions (*b* = -0.06, *SE* = 0.077, *t*(213) = -0.81, *p* = .417).

**Table 1S**

*Correlations Between Raw Personal and Others’ Values in Study 1 (USA)*

| Personal values | Others’ values | | | | | | | | | |
| --- | --- | --- | --- | --- | --- | --- | --- | --- | --- | --- |
|  | 1. | 2. | 3. | 4. | 5. | 6. | 7. | 8. | 9. | 10. |
| 1. Benevolence | .**44**** | .22** | .34** | .31** | .34** | .37** | .25** | .39** | .25** | .25** |
| 2. Universalism | .35** | **.34**** | .36** | .39** | .34** | .39** | .26** | .41** | .29** | .32** |
| 3. Self-direction | .43** | .28** | **.45**** | .32** | .38** | .37** | .23** | .46** | .25** | .28** |
| 4. Stimulation | .34** | .38** | .28** | **.50**** | .35** | .33** | .33** | .29** | .40** | .41** |
| 5. Hedonism | .34** | .33** | .30** | .43** | **.43**** | .30** | .25** | .34** | .31** | .35** |
| 6. Achievement | .39** | .44** | .36** | .45** | .36** | **.41**** | .30** | .31** | .44** | .43** |
| 7. Power | .31** | .43** | .25** | .33** | .16** | .25** | **.38**** | .20** | .49** | .46** |
| 8. Security | .46** | .34** | .47** | .36** | .35** | .31** | .16** | **.54**** | .32** | .33** |
| 9. Conformity | .39** | .40** | .42** | .41** | .27** | .26** | .20** | .35** | **.49**** | .45** |
| 10. Tradition | .46** | .43** | .41** | .43** | .32** | .30** | .19** | .35** | .46** | **.51**** |

*Notes*. *N* = 361. * *p* < .05, ** *p* < .01.

**Table 2S**

*Correlations Between Raw Personal and Others’ Values in Study 1 (China)*

| Personal values | Others’ values | | | | | | | | | |
| --- | --- | --- | --- | --- | --- | --- | --- | --- | --- | --- |
|  | 1. | 2. | 3. | 4. | 5. | 6. | 7. | 8. | 9. | 10. |
| 1. Benevolence | **.66**** | .56** | .50** | .53** | .52** | .59** | .48** | .52** | .53** | .50** |
| 2. Universalism | .61** | **.60**** | .49** | .54** | .55** | .51** | .42** | .48** | .51** | .49** |
| 3. Self-direction | .49** | .41** | **.48**** | .43** | .51** | .49** | .39** | .51** | .40** | .33** |
| 4. Stimulation | .48** | .47** | .40** | **.60**** | .49** | .47** | .44** | .31** | .42** | .39** |
| 5. Hedonism | .53** | .49** | .51** | .51** | **.56**** | .51** | .48** | .39** | .45** | .38** |
| 6. Achievement | .56** | .45** | .43** | .51** | .53** | **.62**** | .58** | .35** | .43** | .42** |
| 7. Power | .48** | .47** | .42** | .52** | .38** | .54** | **.62**** | .20** | .42** | .46** |
| 8. Security | .60** | .53** | .55** | .46** | .56** | .49** | .41** | **.68**** | .52** | .46** |
| 9. Conformity | .57** | .53** | .46** | .48** | .50** | .51** | .46** | .48** | **.56**** | .53** |
| 10. Tradition | .47** | .51** | .37** | .47** | .31** | .38** | .30** | .31** | .50** | **.65**** |

*Notes*. *N* = 286. * *p* < .05, ** *p* < .01.

**Table 3S**

*Correlations Between Raw Personal and Others’ Values in Study 1 (Malaysia)*

| Personal values | Others’ values | | | | | | | | | |
| --- | --- | --- | --- | --- | --- | --- | --- | --- | --- | --- |
|  | 1. | 2. | 3. | 4. | 5. | 6. | 7. | 8. | 9. | 10. |
| 1. Benevolence | **.51**** | .39** | .42** | .40** | .36** | .48** | .24** | .45** | .39** | .42** |
| 2. Universalism | .49** | **.48**** | .49** | .46** | .34** | .47** | .25** | .43** | .43** | .46** |
| 3. Self-direction | .44** | .40** | **.50**** | .43** | .43** | .42** | .37** | .37** | .38** | .42** |
| 4. Stimulation | .39** | .36** | .40** | **.45**** | .39** | .38** | .25** | .37** | .34** | .39** |
| 5. Hedonism | .35** | .32** | .33** | .38** | **.51**** | .35** | .29** | .25** | .29** | .43** |
| 6. Achievement | .44** | .36** | .33** | .35** | .31** | **.47**** | .26** | .36** | .37** | .42** |
| 7. Power | .22** | .27** | .25** | .29** | .27** | .24** | **.47**** | .09 | .25** | .35** |
| 8. Security | .47** | .41** | .44** | .40** | .34** | .47** | .21** | **.52**** | .40** | .37** |
| 9. Conformity | .47** | .47** | .46** | .47** | .26** | .49** | .24** | .46** | **.48**** | .43** |
| 10. Tradition | .46** | .43** | .46** | .49** | .35** | .46** | .31** | .38** | .41** | **.51**** |

*Notes*. *N* = 346. * *p* < .05, ** *p* < .01.

**Table 4S**

*Correlations Between Raw Personal Values and Self-Others’ Differences in Study 1 (USA)*

| Personal values | Self-others’ differences | | | | | | | | | |
| --- | --- | --- | --- | --- | --- | --- | --- | --- | --- | --- |
|  | 1. | 2. | 3. | 4. | 5. | 6. | 7. | 8. | 9. | 10. |
| 1. Benevolence | **.41^**^** | .28^**^ | .17^**^ | .04 | .10^*^ | .09 | -.15^**^ | .23^**^ | .26^**^ | .22^**^ |
| 2. Universalism | .20^**^ | **.45^**^** | .16^**^ | .13^*^ | .15^**^ | .09 | -.11^*^ | .14^**^ | .23^**^ | .12^*^ |
| 3. Self-direction | .11^*^ | .23^**^ | **.36^**^** | .13^*^ | .11^*^ | .12^*^ | -.09 | .11^*^ | .10 | .11^*^ |
| 4. Stimulation | -.07 | .01 | .05 | **.55^**^** | .35^**^ | .34^**^ | .15^**^ | -.05 | -.11^*^ | -.10 |
| 5. Hedonism | .02 | .06 | .08 | .30^**^ | **.56^**^** | .32^**^ | .09 | .04 | .03 | -.03 |
| 6. Achievement | -.04 | -.08 | -.01 | .24^**^ | .24^**^ | **.62^**^** | .26^**^ | .03 | -.10 | -.11^*^ |
| 7. Power | -.26^**^ | -.31^**^ | -.16^**^ | .18^**^ | .17^**^ | .34^**^ | **.59^**^** | -.10 | -.27^**^ | -.25^**^ |
| 8. Security | .10 | .15^**^ | .03 | -.06 | .08 | .10^*^ | -.03 | **.39^**^** | .27^**^ | .18^**^ |
| 9. Conformity | .07 | .05 | -.12^*^ | -.06 | .10 | .13^*^ | .05 | .24^**^ | **.45^**^** | .19^*^ |
| 10. Tradition | -.01 | -.01 | -.06 | -.02 | .06 | .12^*^ | .09 | .18^**^ | .16^**^ | **.36^**^** |

*Notes*. *N* = 361. * *p* < .05, ** *p* < .01.

**Table 5S**

*Correlations Between Raw Personal Values and Self-Others’ Differences in Study 1 (China)*

| Personal values | Self-others’ differences | | | | | | | | | |
| --- | --- | --- | --- | --- | --- | --- | --- | --- | --- | --- |
|  | 1. | 2. | 3. | 4. | 5. | 6. | 7. | 8. | 9. | 10. |
| 1. Benevolence | **.44**** | .14* | .08 | .06 | .08 | .13* | .10 | .19** | .14* | .07 |
| 2. Universalism | .24** | **.33**** | .14* | .15* | .08 | .18** | .13* | .28** | .16** | .10 |
| 3. Self-direction | .22** | .26** | **.41**** | .25** | .16** | .17** | .03 | .23** | .15* | .10 |
| 4. Stimulation | .10 | .11 | .15** | **.49**** | .18** | .22** | .20** | .09 | -.08 | .01 |
| 5. Hedonism | .13* | .09 | .09 | .21** | **.44**** | .24** | .12* | .21** | -.01 | -.06 |
| 6. Achievement | .10 | .10 | .07 | .13* | .10 | **.54**** | .21** | .22** | .07 | -.04 |
| 7. Power | .07 | -.05 | -.09 | .08 | .16** | .28** | **.52**** | .22** | -.05 | -.07 |
| 8. Security | .13* | .13* | .09 | -.05 | .00 | .13* | .01 | **.35**** | .16** | .05 |
| 9. Conformity | .21** | .13* | .06 | -.09 | -.02 | .11 | -.01 | .28** | **.38**** | .15* |
| 10. Tradition | .13* | .01 | .00 | -.05 | .01 | .07 | .16** | .22** | .12 | **.40**** |

*Notes*. *N* = 286. * *p* < .05, ** *p* < .01.

**Table 6S**

*Correlations Between Raw Personal Values and Self-Others’ Differences in Study 1 (Malaysia)*

| Personal values | Self-others’ differences | | | | | | | | | |
| --- | --- | --- | --- | --- | --- | --- | --- | --- | --- | --- |
|  | 1. | 2. | 3. | 4. | 5. | 6. | 7. | 8. | 9. | 10. |
| 1. Benevolence | **.46**** | .23** | .23** | .22** | .25** | .23** | .09 | .29** | .19** | .16** |
| 2. Universalism | .22** | **.38**** | .17** | .18** | .17** | .19** | .17** | .24** | .19** | .21** |
| 3. Self-direction | .21** | .17** | **.48**** | .20** | .15** | .22** | .14* | .22** | .09 | .14* |
| 4. Stimulation | .21** | .18** | .21** | **.55**** | .22** | .26** | .19** | .14* | .07 | .11* |
| 5. Hedonism | .20** | .07 | .19** | .18** | **.57**** | .24** | .25** | .17** | -.02 | .01 |
| 6. Achievement | .21** | .17** | .27** | .25** | .29** | **.57**** | .23** | .22** | .09 | .06 |
| 7. Power | .09 | .07 | .23** | .14** | .30** | .25** | **.55**** | .14** | -.02 | .00 |
| 8. Security | .30** | .21** | .18** | .15** | .15** | .18** | .06 | **.43**** | .20** | .19** |
| 9. Conformity | .21** | .16** | .10 | .04 | .11* | .10 | .06 | .21** | **.36**** | .17** |
| 10. Tradition | .20** | .22** | .18** | .11* | .21** | .14** | .12* | .22** | .16** | **.37**** |

*Notes*. *N* = 346. * *p* < .05, ** *p* < .01.

**Table 7S**

*Correlations Between Raw Personal and Others’ Values in Study 2 (USA)*

| Personal values | Others’ values | | | | | | | | | |
| --- | --- | --- | --- | --- | --- | --- | --- | --- | --- | --- |
|  | 1. | 2. | 3. | 4. | 5. | 6. | 7. | 8. | 9. | 10. |
| 1. Benevolence | **.40**** | .20** | .36** | .18** | .27** | .28** | .09 | .30** | .17** | .13* |
| 2. Universalism | .27** | **.26**** | .29** | .18** | .16** | .20** | .03 | .21** | .22** | .17** |
| 3. Self-direction | .24** | .01 | **.21**** | .04 | .25** | .30** | .14** | .37** | .08 | .11* |
| 4. Stimulation | .11* | .12* | .05 | **.17**** | .20** | .15** | .15** | .12* | .19** | .25** |
| 5. Hedonism | .21** | .13* | .17** | .16** | **.30**** | .22** | .15** | .24** | .18** | .26** |
| 6. Achievement | .09 | .11* | .14** | .25** | .26** | **.28**** | .21** | .15** | .18** | .24** |
| 7. Power | -.05 | .16** | .00 | .23** | .13* | .06 | **.25**** | -.06 | .16** | .20** |
| 8. Security | .27** | .24** | .38** | .23** | .21** | .28** | .10* | **.47**** | .19** | .16** |
| 9. Conformity | .21** | .32** | .32** | .29** | .12* | .12* | .01 | .13* | **.29**** | .12* |
| 10. Tradition | .24** | .38** | .34** | .25** | .11* | .08 | -.01 | .13* | .23** | **.24**** |

*Notes*. *N* = 363. * *p* < .05, ** *p* < .01.

**Table 8S**

*Correlations Between Raw Personal Values and Self-Others’ Differences in Study 2 (USA)*

| Personal values | Self-others’ differences | | | | | | | | | |
| --- | --- | --- | --- | --- | --- | --- | --- | --- | --- | --- |
|  | 1. | 2. | 3. | 4. | 5. | 6. | 7. | 8. | 9. | 10. |
| 1. Benevolence | **.61**** | .25** | .00 | .08 | .00 | .04 | -.14** | .24** | .19** | .33** |
| 2. Universalism | .24** | **.61**** | .11* | .17** | .04 | .01 | -.10 | .06 | .03 | .10 |
| 3. Self-direction | .17** | .37** | **.57**** | .29** | .11* | .02 | -.19** | -.04 | -.12* | -.05 |
| 4. Stimulation | .11* | .16** | .22** | **.78**** | .43** | .38** | .18** | -.09 | -.22** | -.06 |
| 5. Hedonism | .04 | .05 | .11* | .47** | **.68**** | .23** | .09 | -.10* | -.24** | -.16** |
| 6. Achievement | .17** | .05 | .07 | .31** | .18** | **.72**** | .34** | .15** | -.04 | -.03 |
| 7. Power | -.03 | -.20** | -.08 | .15** | .11* | .48** | **.69**** | .22** | -.08 | -.05 |
| 8. Security | .21** | -.01 | -.12* | -.14** | -.08 | .04 | .06 | **.65**** | .27** | .35** |
| 9. Conformity | .17** | -.08 | -.32** | -.27** | -.19** | .02 | .03 | .37** | **.68**** | .45** |
| 10. Tradition | .23** | -.12* | -.26** | -.06 | -.07 | .07 | .09 | .44** | .35** | **.74**** |

*Notes*. *N* = 363. * *p* < .05, ** *p* < .01.

**Table 9S**

*Correlations Between Raw Personal and Others’ Values in Study 3 (Israel)*

| Personal values | Others’ values | | | | | | | | | |
| --- | --- | --- | --- | --- | --- | --- | --- | --- | --- | --- |
|  | 1. | 2. | 3. | 4. | 5. | 6. | 7. | 8. | 9. | 10. |
| 1. Benevolence | **.46**** | .40** | .49** | .25** | .14* | .31** | .27** | .40** | .49** | .36** |
| 2. Universalism | .32** | **.45**** | .39** | .19** | .20** | .20** | .14* | .34** | .46** | .45** |
| 3. Self-direction | .31** | .37** | **.45**** | .16* | .23** | .26** | .25** | .38** | .47** | .42** |
| 4. Stimulation | .23** | .32** | .25** | **.16*** | .21** | .09 | .19** | .33** | .48** | .39** |
| 5. Hedonism | .10 | .17* | .14* | -.01 | **.38**** | .03 | .05 | .19** | .27** | .21** |
| 6. Achievement | .30** | .33** | .39** | .20** | .11 | **.35**** | .20** | .24** | .44** | .35** |
| 7. Power | .13 | .06 | .06 | .08 | .16* | .06 | **.39**** | .16* | .27** | .19** |
| 8. Security | .28** | .38** | .38** | .23** | .30** | .17* | .29** | **.49**** | .47** | .35** |
| 9. Conformity | .31** | .40** | .52** | .28** | .25** | .26** | .35** | .40** | **.57**** | .43** |
| 10. Tradition | .30** | .46** | .38** | .29** | .22** | .15* | .14* | .38** | .47** | **.47**** |

*Notes*. *N* = 227. * *p* < .05, ** *p* < .01.

**Table 10S**

*Correlations Between Raw Personal Values and Self-Others’ Differences in Study 3 (Israel)*

| Personal values | Self-others’ differences | | | | | | | | | |
| --- | --- | --- | --- | --- | --- | --- | --- | --- | --- | --- |
|  | 1. | 2. | 3. | 4. | 5. | 6. | 7. | 8. | 9. | 10. |
| 1. Benevolence | **.44**** | .05 | -.05 | .04 | -.05 | .10 | -.08 | .16* | .19** | .24** |
| 2. Universalism | .13 | **.45**** | .16* | .25** | .11 | .08 | -.01 | .04 | .01 | .08 |
| 3. Self-direction | .10 | .16* | **.48**** | .40** | .21** | .27** | .14* | .11 | .05 | -.07 |
| 4. Stimulation | .00 | .09 | .33** | **.78**** | .37** | .31** | .26** | .07 | -.09 | -.13 |
| 5. Hedonism | -.04 | .09 | .25** | .48** | **.64**** | .23** | .36** | .14* | -.08 | -.20** |
| 6. Achievement | .08 | -.06 | .13 | .22** | .19** | **.58**** | .37** | .27** | .09 | .01 |
| 7. Power | .01 | .05 | .28** | .31** | .27** | .45** | **.62**** | .33** | .14* | -.01 |
| 8. Security | .18** | -.06 | .04 | .14* | .04 | .29** | .19** | **.57**** | .30** | .27** |
| 9. Conformity | .25** | .00 | -.08 | .10 | -.04 | .21** | .06 | .35** | **.53**** | .30** |
| 10. Tradition | .16* | -.05 | -.11 | -.04 | -.19** | .13* | .02 | .20** | .22** | **.65**** |

*Notes*. *N* = 227. * *p* < .05, ** *p* < .01.

**Table 11S**

*Means, Standard Deviations, and Ranks of Ipsatized Personal and Others’ Values in Study 1 (USA)*

| Values | Personal | | | | Others | | | |
| --- | --- | --- | --- | --- | --- | --- | --- | --- |
|  | Rank | *M* | *SD* | Cronbach’s alpha | Rank | *M* | *SD* | Cronbach’s alpha |
| Benevolence | 1 | 0.61 | 0.55 | .86 | 5 | 0.18 | 0.49 | .91 |
| Universalism | 4 | 0.21 | 0.55 | .76 | 8 | -0.22 | 0.62 | .80 |
| Self-direction | 2 | 0.51 | 0.55 | .80 | 3 | 0.28 | 0.56 | .81 |
| Stimulation | 9 | -0.37 | 0.80 | .72 | 6 | -0.06 | 0.65 | .71 |
| Hedonism | 5 | 0.10 | 0.73 | .90 | 2 | 0.35 | 0.74 | .90 |
| Achievement | 8 | -0.21 | 0.75 | .81 | 4 | 0.22 | 0.71 | .88 |
| Power | 10 | -1.46 | 1.11 | .87 | 7 | -0.29 | 1.14 | .91 |
| Security | 3 | 0.49 | 0.52 | .76 | 1 | 0.41 | 0.55 | .87 |
| Conformity | 7 | -0.10 | 0.71 | .90 | 9 | -0.33 | 0.70 | .94 |
| Tradition | 6 | -0.08 | 0.61 | .84 | 10 | -0.31 | 0.60 | .89 |

*Note*. *N* = 361.

**Table 12S**

*Means, Standard Deviations, and Ranks of Ipsatized Personal and Others’ Values in Study 1 (China)*

| Value | Personal | | | | Others | | | |
| --- | --- | --- | --- | --- | --- | --- | --- | --- |
|  | Rank | *M* | *SD* | Cronbach’s alpha | Rank | *M* | *SD* | Cronbach’s alpha |
| Benevolence | 3 | 0.24 | 0.42 | .88 | 3 | 0.11 | 0.35 | .91 |
| Universalism | 4 | 0.13 | 0.38 | .75 | 6 | -0.13 | 0.43 | .80 |
| Self-direction | 2 | 0.36 | 0.49 | .65 | 4 | 0.03 | 0.45 | .77 |
| Stimulation | 9 | -0.37 | 0.74 | .78 | 10 | -0.31 | 0.57 | .73 |
| Hedonism | 5 | 0.06 | 0.58 | .83 | 2 | 0.18 | 0.63 | .84 |
| Achievement | 7 | -0.21 | 0.67 | .86 | 5 | 0.02 | 0.60 | .87 |
| Power | 10 | -0.83 | 0.73 | .84 | 7 | -0.16 | 0.75 | .88 |
| Security | 1 | 0.48 | 0.48 | .81 | 1 | 0.56 | 0.59 | .84 |
| Conformity | 6 | 0.06 | 0.48 | .91 | 9 | -0.12 | 0.45 | .93 |
| Tradition | 8 | -0.25 | 0.63 | .87 | 7 | -0.25 | 0.52 | .87 |

*Note*. *N* = 286.

**Table 13S**

*Means, Standard Deviations, and Ranks of Ipsatized Personal and Others’ Values in Study 1 (Malaysia)*

| Value | Personal | | | | Others | | | |
| --- | --- | --- | --- | --- | --- | --- | --- | --- |
|  | Rank | *M* | *SD* | Cronbach’s alpha | Rank | *M* | *SD* | Cronbach’s alpha |
| Benevolence | 2 | 0.35 | 0.39 | .80 | 3 | 0.19 | 0.35 | .87 |
| Universalism | 6 | 0.05 | 0.35 | .73 | 8 | -0.09 | 0.43 | .70 |
| Self-direction | 5 | 0.06 | 0.44 | .67 | 4 | 0.04 | 0.37 | .66 |
| Stimulation | 8 | -0.07 | 0.58 | .68 | 7 | -0.09 | 0.49 | .63 |
| Hedonism | 9 | -0.25 | 0.70 | .81 | 5 | -0.03 | 0.68 | .83 |
| Achievement | 4 | 0.12 | 0.51 | .83 | 2 | 0.19 | 0.49 | .90 |
| Power | 10 | -1.16 | 0.82 | .81 | 10 | -0.42 | 0.87 | .91 |
| Security | 1 | 0.49 | 0.42 | .71 | 1 | 0.39 | 0.46 | .84 |
| Conformity | 3 | 0.24 | 0.47 | .84 | 6 | -0.08 | 0.51 | .91 |
| Tradition | 7 | -0.01 | 0.41 | .82 | 9 | -0.13 | 0.44 | .87 |

*Note*. *N* = 346.

**Table 14S**

*Correlations Between Ipsatized Personal and Others’ Values in Study 1 (USA)*

| Personal values | Others’ values | | | | | | | | | |
| --- | --- | --- | --- | --- | --- | --- | --- | --- | --- | --- |
|  | 1. | 2. | 3. | 4. | 5. | 6. | 7. | 8. | 9. | 10. |
| 1. Benevolence | **.28**** | -.26** | .07 | -.05 | .17** | .22** | .06 | .21** | -.25** | -.25** |
| 2. Universalism | -.07 | **-.04** | .02 | .05 | .10 | .18** | .04 | .19** | -.21** | -.16** |
| 3. Self-direction | .16** | -.19** | **.25**** | -.11* | .19** | .15** | .00 | .29** | -.30** | -.24** |
| 4. Stimulation | -.13* | .04 | -.21** | **.24**** | .04 | .00 | .10 | -.19** | .04 | .07 |
| 5. Hedonism | -.05 | -.02 | -.09 | .16** | **.25**** | .01 | .03 | .00 | -.09 | -.03 |
| 6. Achievement | -.09 | .11* | -.10 | .08 | .01 | **.07** | .02 | -.22** | .07 | .05 |
| 7. Power | -.16** | .17** | -.23** | -0.08 | -.24** | -.12* | **.15**** | -.32** | .24** | .19** |
| 8. Security | .20** | -.10 | .26** | -.08 | .08 | -.02 | -.15** | **.45**** | -.19** | -.20** |
| 9. Conformity | -.06 | .06 | .08 | .01 | -.13* | -.17** | -.11* | -.11* | **.25**** | .13* |
| 10. Tradition | .09 | .07 | .02 | .02 | -.05 | -.11* | -.15** | -.13* | .10 | **.23**** |

*Notes*. *N* = 361. * *p* < .05, ** *p* < .01.

**Table 15S**

*Correlations Between Ipsatized Personal and Others’ Values in Study 1 (China)*

| Personal values | Others’ values | | | | | | | | | |
| --- | --- | --- | --- | --- | --- | --- | --- | --- | --- | --- |
|  | 1. | 2. | 3. | 4. | 5. | 6. | 7. | 8. | 9. | 10. |
| 1. Benevolence | **.21**** | .04 | -.07 | -.09 | -.05 | .07 | -.07 | .08 | .00 | -.10 |
| 2. Universalism | .10 | **.26**** | -.03 | .01 | .10 | -.11 | -.20** | .04 | -.03 | -.04 |
| 3. Self-direction | -.04 | -.21** | **.22**** | -.09 | .17** | .05 | -.03 | .32** | -.11 | -.25** |
| 4. Stimulation | -.04 | .04 | -.04 | **.38**** | .12* | .03 | .05 | -.17** | -.04 | -.08 |
| 5. Hedonism | -.03 | -.03 | .17** | .07 | **.18**** | .02 | .08 | -.06 | -.05 | -.21** |
| 6. Achievement | .06 | -.16** | -.09 | .08 | .12* | **.29**** | .25** | -.20** | -.14* | -.11 |
| 7. Power | -.09 | .00 | -.02 | .14* | -.13* | .15** | **.34**** | -.42** | -.07 | .06 |
| 8. Security | .01 | -.10 | .14* | -.25** | .07 | -.18** | -.20** | **.53**** | -.01 | -.17** |
| 9. Conformity | -.05 | -.04 | -.10 | -.131* | -.04 | -.08 | -.04 | .07 | **.21**** | .10 |
| 10. Tradition | -.09 | .14* | -.13* | .04 | -.24** | -.17** | -.18** | -.12* | .19** | **.55**** |

*Notes*. *N* = 286. * *p* < .05, ** *p* < .01.

**Table 16S**

*Correlations Between Ipsatized Personal and Others’ Values in Study 1 (Malaysia)*

| Personal values | Others’ values | | | | | | | | | |
| --- | --- | --- | --- | --- | --- | --- | --- | --- | --- | --- |
|  | 1. | 2. | 3. | 4. | 5. | 6. | 7. | 8. | 9. | 10. |
| 1. Benevolence | **.28**** | -.08 | -.06 | -.10 | -.01 | .11* | -.15** | .23** | -.03 | -.06 |
| 2. Universalism | .08 | **.19**** | .11* | .02 | -.12* | .00 | -.20** | .09 | .03 | -.03 |
| 3. Self-direction | -.06 | -.09 | **.23**** | -.03 | .12* | -.09 | .10 | -.07 | -.08 | -.10 |
| 4. Stimulation | -.01 | -.03 | .05 | **.16**** | .12* | -.04 | -.05 | .06 | -.05 | .00 |
| 5. Hedonism | -.07 | -.05 | -.08 | .05 | **.34**** | -.05 | .04 | -.18** | -.09 | .18** |
| 6. Achievement | .16** | -.03 | -.19** | -.09 | -.03 | **.18**** | -.04 | .03 | .03 | .08 |
| 7. Power | -.27** | -.02 | -.12* | .00 | .04 | -.11* | **.37**** | -.36** | -.06 | .11* |
| 8. Security | .13* | -.01 | .03 | -.08 | -.05 | .08 | -.19** | **.41**** | .02 | -.23** |
| 9. Conformity | -.01 | .12* | .01 | .05 | -.27** | .05 | -.16** | .14** | **.18**** | -.09 |
| 10. Tradition | -.02 | .00 | .01 | .13* | -.07 | -.02 | -.04 | -.07 | .00 | **.18**** |

*Notes*. *N* = 346. * *p* < .05, ** *p* < .01.

**Table 17S**

*Correlations Between Ipsatized Personal Values and Self-Others’ Differences in Study 1 (USA)*

| Personal values | Self-others’ differences | | | | | | | | | |
| --- | --- | --- | --- | --- | --- | --- | --- | --- | --- | --- |
|  | 1. | 2. | 3. | 4. | 5. | 6. | 7. | 8. | 9. | 10. |
| 1. Benevolence | **.65**** | .28** | .15** | -.25** | -.24** | -.32** | -.42** | .04 | .15** | .19** |
| 2. Universalism | .17** | **.68**** | .12* | -.12* | -.18** | -.35** | -.40** | -.16** | .10 | -.03 |
| 3. Self-direction | .10 | .24** | **.60**** | -0.03 | -.15** | -.21** | -.28** | -.08 | -.08 | .07 |
| 4. Stimulation | -.18** | -.09 | .07 | **.72**** | .33** | .26** | .09 | -.26** | -.37** | -.23** |
| 5. Hedonism | -.07 | -.06 | .08 | .28** | **.60**** | .16** | -.02 | -.19** | -.17** | -.18** |
| 6. Achievement | -.12* | -.27** | -.03 | .25** | .18** | **.70**** | .25** | -.12* | -.33** | -.25** |
| 7. Power | -.30** | -.44** | -.11* | .26** | .20** | .35** | **.64**** | -.10 | -.38** | -.27** |
| 8. Security | .07 | .08 | -.04 | -.36** | -.23** | -.26** | -.22** | **.50**** | .24** | .20** |
| 9. Conformity | -.01 | -.10 | -.38** | -.33** | -.13* | -.13* | -.08 | .21** | **.61**** | .08 |
| 10. Tradition | -.08 | -.18** | -.15** | -.20** | -.16** | -.13* | .00 | .16** | .10 | **.62**** |

*Notes*. *N* = 361. * *p* < .05, ** *p* < .01.

**Table 18S**

*Correlations Between Ipsatized Personal Values and Self-Others’ Differences in Study 1 (China)*

| Personal values | Self-others’ differences | | | | | | | | | |
| --- | --- | --- | --- | --- | --- | --- | --- | --- | --- | --- |
|  | 1. | 2. | 3. | 4. | 5. | 6. | 7. | 8. | 9. | 10. |
| 1. Benevolence | **.70**** | .01 | -.06 | -.10 | -.05 | -.18** | -.13* | -.16** | .03 | -.06 |
| 2. Universalism | -.02 | **.54**** | .02 | .04 | -.147* | -.16** | -.17** | -.07 | -.02 | -.09 |
| 3. Self-direction | -.09 | .19** | **.67**** | .22** | .03 | -.15* | -.30** | -.17** | -.05 | -.08 |
| 4. Stimulation | -.12* | .00 | .16** | **.70**** | .14* | .09 | .09 | -.20** | -.37** | -.12* |
| 5. Hedonism | -.08 | -.04 | .05 | .26** | **.60**** | .10 | -.02 | -.02 | -.24** | -.23** |
| 6. Achievement | -.17** | -.07 | -.03 | .07 | .00 | **.65**** | .10 | .00 | -.11 | -.26** |
| 7. Power | -.12* | -.30** | -.30** | .04 | .15* | .21** | **.57**** | .08 | -.24** | -.20** |
| 8. Security | -.07 | .06 | .06 | -.24** | -.13* | -.08 | -.21** | **.31**** | .17** | .01 |
| 9. Conformity | .07 | .00 | -.07 | -.39** | -.22** | -.17** | -.28** | .10 | **.65**** | .17** |
| 10. Tradition | -.05 | -.20** | -.13* | -.20** | -.08 | -.15* | .04 | .02 | .05 | **.62**** |

*Notes*. *N* = 286. * *p* < .05, ** *p* < .01.

**Table 19S**

*Correlations Between Ipsatized Personal Values and Self-Others’ Differences in Study 1 (Malaysia)*

| Personal values | Self-others’ differences | | | | | | | | | |
| --- | --- | --- | --- | --- | --- | --- | --- | --- | --- | --- |
|  | 1. | 2. | 3. | 4. | 5. | 6. | 7. | 8. | 9. | 10. |
| 1. Benevolence | **.65**** | .02 | -.07 | .03 | -.02 | -.06 | -.27** | .07 | .06 | -.05 |
| 2. Universalism | -.12* | **.55**** | -.27** | -.06 | -.19** | -.14* | -.11* | -.07 | .10 | .12* |
| 3. Self-direction | -.07 | -.08 | **.70**** | .04 | -.15** | -.01 | -.11* | -.05 | -.11* | -.02 |
| 4. Stimulation | -.04 | -.02 | -.01 | **.72**** | -.01 | .08 | .01 | -.22** | -.14* | -.08 |
| 5. Hedonism | .03 | -.19** | .01 | .06 | **.59**** | .09 | .13* | -.05 | -.25** | -.23** |
| 6. Achievement | -.10 | -.10 | .06 | .12* | .08 | **.66**** | .04 | -.07 | -.14** | -.24** |
| 7. Power | -.25** | -.22** | .07 | -.03 | .14** | .09 | **.52**** | -.126* | -.25** | -.25** |
| 8. Security | .18** | .02 | -.13* | -.10 | -.18** | -.10 | -.28** | **.49**** | .15** | .10 |
| 9. Conformity | .06 | .02 | -.21** | -.24** | -.15** | -.17** | -.20** | .06 | **.60**** | .15** |
| 10. Tradition | -.10 | .09 | -.12* | -.17** | -.04 | -.18** | -.15** | -.03 | .08 | **.61**** |

*Notes*. *N* = 346. * *p* < .05, ** *p* < .01.

**Table 20S**

*Pairwise Comparisons Between Ipsatized Personal and Others’ Values in the Three Value Priority Groups (High, Middle, Low) in Study 1 (USA)*

| Value | Sample rank | Low Priority Group | | | | | | | | Middle Priority Group | | | | | | | | High Priority Group | | | | | | | | Interaction effect  *F*(2, 358) | | η^2^_partial_ | |  |
| --- | --- | --- | --- | --- | --- | --- | --- | --- | --- | --- | --- | --- | --- | --- | --- | --- | --- | --- | --- | --- | --- | --- | --- | --- | --- | --- | --- | --- | --- | --- |
|  |  | Mean Diff. | | *SE* | | *n* | | *d* | | Mean Diff. | | *SE* | | *n* | | *d* | | Mean Diff. | | *SE* | | *n* | | *d* | |  |  |  |  |  |
| Benevolence | 1 | | 0.02 | | 0.04 | | 120 | | **0.05** | | 0.41*** | | 0.04 | | 122 | | **0.92** | | 0.85*** | | 0.06 | | 119 | | **1.27** | | 74.11*** | | .293 | |
| Universalism | 4 | | -0.12* | | 0.05 | | 120 | | **-0.21** | | 0.33*** | | 0.05 | | 122 | | **0.66** | | 1.09*** | | 0.09 | | 119 | | **1.16** | | 94.13*** | | .345 | |
| Self-direction | 2 | | -0.16*** | | 0.05 | | 118 | | **-0.32** | | 0.20*** | | 0.04 | | 123 | | **0.43** | | 0.66*** | | 0.07 | | 120 | | **0.85** | | 56.58*** | | .240 | |
| Stimulation | 9 | | -1.09*** | | 0.08 | | 120 | | **-1.22** | | -0.22*** | | 0.04 | | 121 | | **-0.46** | | 0.38*** | | 0.05 | | 120 | | **0.64** | | 141.92*** | | .442 | |
| Hedonism | 5 | | -0.85*** | | 0.09 | | 121 | | **-0.82** | | -0.13* | | 0.05 | | 118 | | **-0.22** | | 0.23*** | | 0.06 | | 122 | | **0.38** | | 62.17*** | | .258 | |
| Achievement | 8 | | -1.24*** | | 0.10 | | 120 | | **-1.18** | | -0.31*** | | 0.06 | | 121 | | **-0.51** | | 0.24*** | | 0.06 | | 120 | | **0.37** | | 105.47*** | | .371 | |
| Power | 10 | | -2.16*** | | 0.15 | | 120 | | **-1.31** | | -1.24*** | | 0.10 | | 120 | | **-1.08** | | -0.12* | | 0.05 | | 121 | | **-0.19** | | 86.56*** | | .326 | |
| Security | 3 | | -0.23*** | | 0.04 | | 120 | | **-0.47** | | 0.05 | | 0.04 | | 120 | | **0.11** | | 0.40*** | | 0.05 | | 121 | | **0.72** | | 46.98*** | | .208 | |
| Conformity | 7 | | -0.29*** | | 0.07 | | 119 | | **-0.40** | | 0.19*** | | 0.05 | | 123 | | **0.35** | | 0.78*** | | 0.09 | | 119 | | **0.84** | | 143.28*** | | .254 | |
| Tradition | 6 | | -0.22*** | | 0.06 | | 120 | | **-0.34** | | 0.15*** | | 0.04 | | 121 | | **0.34** | | 0.78*** | | 0.07 | | 120 | | **1.02** | | 77.27*** | | .302 | |

*Notes*. *N* = 361. Mean Difference is calculated as personal values minus others’ values. * *p* < .05, ** *p* < .01, *** *p* < .001.

**Table 21S**

*Pairwise Comparisons Between Ipsatized Personal and Others’ Values in the Three Value Priority Groups (High, Middle, Low) in Study 1 (China)*

| Value | Sample rank | Low Priority Group | | | | Middle Priority Group | | | | High Priority Group | | | | Interaction effect  *F*(2, 283) | η^2^_partial_ |
| --- | --- | --- | --- | --- | --- | --- | --- | --- | --- | --- | --- | --- | --- | --- | --- |
|  |  | Mean Diff. | *SE* | *n* | *d* | Mean Diff. | *SE* | *N* | *d* | Mean Diff. | *SE* | *n* | *d* |  |  |
| Benevolence | 3 | -0.25*** | 0.05 | 95 | **-0.54** | 0.20*** | 0.03 | 94 | **0.39** | 0.44*** | 0.04 | 97 | **1.14** | 75.94*** | .349 |
| Universalism | 4 | 0.01 | 0.04 | 96 | **0.02** | 0.19*** | 0.03 | 95 | **0.60** | 0.59*** | 0.05 | 95 | **1.14** | 45.01*** | .241 |
| Self-direction | 2 | -0.07^a^ | 0.04 | 95 | **-0.19** | 0.32*** | 0.04 | 96 | **0.84** | 0.73*** | 0.07 | 95 | **1.12** | 63.72*** | .310 |
| Stimulation | 9 | -0.59*** | 0.06 | 95 | **-0.93** | -0.06 | 0.05 | 96 | **-0.13** | 0.49*** | 0.07 | 95 | **0.71** | 75.16*** | .347 |
| Hedonism | 5 | -0.63*** | 0.08 | 96 | **-0.76** | 0.00 | 0.05 | 96 | **0.00** | 0.29*** | 0.07 | 94 | **0.44** | 45.99*** | .245 |
| Achievement | 7 | -0.80*** | 0.07 | 94 | **-1.16** | -0.22*** | 0.05 | 97 | **-0.40** | 0.31*** | 0.06 | 95 | **0.53** | 79.44*** | .360 |
| Power | 10 | -1.24*** | 0.10 | 95 | **-1.33** | -0.67*** | 0.07 | 95 | **-0.98** | -0.09^a^ | 0.05 | 96 | **-0.18** | 59.35*** | .295 |
| Security | 1 | -0.23** | 0.05 | 94 | **-0.46** | -0.03 | 0.05 | 98 | **-0.07** | 0.03 | 0.06 | 94 | **0.05** | 6.40** | .043 |
| Conformity | 6 | -0.17*** | 0.05 | 95 | **-0.34** | 0.09* | 0.04 | 95 | **0.26** | 0.61*** | 0.06 | 96 | **1.06** | 63.06*** | .308 |
| Tradition | 8 | -0.36*** | 0.05 | 96 | **-0.69** | -0.02 | 0.03 | 94 | **-0.05** | 0.38*** | 0.05 | 96 | **0.73** | 61.21*** | .302 |

*Notes*. *N* = 286. Mean Difference is calculated as personal values minus others’ values. ^a^ *p* < .10, * *p* < .05, ** *p* < .01, *** *p* < .001.

**Table 22S**

*Pairwise Comparisons Between Ipsatized Personal and Others’ Values in the Three Value Priority Groups (High, Middle, Low) in Study 1 (Malaysia)*

| Value | Sample rank | Low Priority Group | | | | Middle Priority Group | | | | High Priority Group | | | | Interaction effect  *F*(2, 343) | η^2^_partial_ |
| --- | --- | --- | --- | --- | --- | --- | --- | --- | --- | --- | --- | --- | --- | --- | --- |
|  |  | Mean Diff. | *SE* | *n* | *d* | Mean Diff. | *SE* | *n* | *d* | Mean Diff. | *SE* | *n* | *d* |  |  |
| Benevolence | 2 | -0.18*** | 0.03 | 115 | **-0.51** | 0.20*** | 0.03 | 115 | **0.61** | 0.47*** | 0.04 | 116 | **1.23** | 98.08*** | .364 |
| Universalism | 6 | -0.14*** | 0.04 | 115 | **-0.33** | 0.17*** | 0.04 | 116 | **0.42** | 0.40*** | 0.05 | 115 | **0.76** | 40.84*** | .192 |
| Self-direction | 5 | -0.36*** | 0.04 | 115 | **-0.76** | 0.07* | 0.03 | 116 | **0.24** | 0.34*** | 0.04 | 115 | **0.73** | 81.67*** | .323 |
| Stimulation | 8 | -0.50*** | 0.05 | 116 | **-0.88** | -0.01 | 0.04 | 115 | **-0.03** | 0.58*** | 0.06 | 115 | **0.97** | 118.57*** | .409 |
| Hedonism | 9 | -0.78*** | 0.08 | 114 | **-0.90** | -0.16** | 0.06 | 116 | **-0.25** | 0.25*** | 0.04 | 116 | **0.55** | 67.83*** | .283 |
| Achievement | 4 | -0.50*** | 0.06 | 115 | **-0.76** | -0.02 | 0.04 | 117 | **-0.05** | 0.31*** | 0.05 | 114 | **0.60** | 62.08*** | .266 |
| Power | 10 | -1.24*** | 0.10 | 113 | **-1.13** | -0.84*** | 0.07 | 118 | **-1.04** | -0.13** | 0.05 | 115 | **-0.27** | 51.52*** | .231 |
| Security | 1 | -0.11** | 0.04 | 116 | **-0.29** | 0.06^a^ | 0.04 | 116 | **0.16** | 0.37*** | 0.05 | 114 | **0.74** | 36.45*** | .175 |
| Conformity | 3 | -0.06 | 0.04 | 115 | **-0.13** | 0.31*** | 0.05 | 117 | **0.63** | 0.74*** | 0.06 | 114 | **1.16** | 62.91*** | .268 |
| Tradition | 7 | -0.22*** | 0.05 | 114 | **-0.45** | 0.10** | 0.04 | 116 | **0.26** | 0.46*** | 0.05 | 116 | **0.89** | 61.68*** | .265 |

*Notes*. *N* = 346. Mean Difference is calculated as personal values minus others’ values. ^a^ *p* < .10, * *p* < .05, ** *p* < .01, *** *p* < .001.

**Table 23S**

*Means, Standard Deviations, and Ranks of Ipsatized Personal and Others’ Values in Study 2 (USA)*

| Value | Personal | | | | Others | | | |
| --- | --- | --- | --- | --- | --- | --- | --- | --- |
|  | Rank | *M* | *SD* | Cronbach’s alpha | Rank | *M* | *SD* | Cronbach’s alpha |
| Benevolence | 2 | 0.75 | 0.59 | .87 | 4 | 0.43 | 0.51 | .87 |
| Universalism | 3 | 0.26 | 0.67 | .84 | 10 | -0.61 | 0.62 | .75 |
| Self-direction | 1 | 0.81 | 0.67 | .82 | 5 | 0.24 | 0.58 | .81 |
| Stimulation | 8 | -0.34 | 1.01 | .71 | 7 | -0.23 | 0.69 | .68 |
| Hedonism | 5 | 0.20 | 0.90 | .88 | 1 | 0.54 | 0.72 | .88 |
| Achievement | 6 | -0.09 | 0.81 | .82 | 3 | 0.50 | 0.64 | .80 |
| Power | 10 | -1.40 | 1.00 | .87 | 6 | -0.09 | 0.92 | .88 |
| Security | 4 | 0.26 | 0.67 | .75 | 2 | 0.51 | 0.56 | .74 |
| Conformity | 7 | -0.20 | 0.84 | .87 | 8 | -0.43 | 0.64 | .91 |
| Tradition | 9 | -0.45 | 0.73 | .88 | 9 | -0.45 | 0.53 | .83 |

*Note*. *N* = 363.

**Table 24S**

*Correlations Between Ipsatized Personal and Others’ Values in Study 2 (USA)*

| Personal values | Others’ values | | | | | | | | | |
| --- | --- | --- | --- | --- | --- | --- | --- | --- | --- | --- |
|  | 1. | 2. | 3. | 4. | 5. | 6. | 7. | 8. | 9. | 10. |
| 1. Benevolence | **.30**** | -.10 | .13* | -.11* | .05 | .07 | -.06 | .09 | -.11* | -.17** |
| 2. Universalism | .11* | **.06** | .05 | -.06 | -.06 | -.01 | -.10 | -.02 | .01 | -.04 |
| 3. Self-direction | .10* | -.27** | **-.03** | -.20** | .10 | .17** | .08 | .27** | -.14* | -.05 |
| 4. Stimulation | -.07 | -.05 | -.21** | **.03** | .08 | .01 | .08 | -.04 | .07 | .18** |
| 5. Hedonism | .02 | -.12* | -.10* | -.05 | **.14**** | .03 | .05 | .05 | -.01 | .11* |
| 6. Achievement | -.17** | -.13* | -.14** | .10 | .12* | **.15**** | .14** | -.05 | .00 | .12* |
| 7. Power | -.24** | .06 | -.22** | .14** | .03 | -.06 | **.23**** | -.23** | .07 | .17** |
| 8. Security | .01 | -.06 | .14** | -.05 | -.06 | .05 | -.07 | **.34**** | -.11* | -.15** |
| 9. Conformity | -.01 | .17** | .14** | .13* | -.11* | -.13* | -.14** | -.12* | **.14**** | -.11* |
| 10. Tradition | .04 | .27** | .14** | .05 | -.15** | -.20** | -.17** | -.16** | .04 | **.07** |

*Notes*. *N* = 363. * *p* < .05, ** *p* < .01.

**Table 25S**

*Correlations Between Ipsatized Personal Values and Self-Others’ Differences in Study 2 (USA)*

| Personal values | Self-others’ differences | | | | | | | | | |
| --- | --- | --- | --- | --- | --- | --- | --- | --- | --- | --- |
|  | 1. | 2. | 3. | 4. | 5. | 6. | 7. | 8. | 9. | 10. |
| 1. Benevolence | **.67**** | .18** | -.01 | -.10 | -.11* | -.26** | -.38** | -.05 | .05 | .15** |
| 2. Universalism | .05 | **.72**** | .15** | .05 | -.04 | -.25** | -.29** | -.28** | -.16** | -.17** |
| 3. Self-direction | .01 | .38** | **.76**** | .23** | .10 | -.17** | -.31** | -.32** | -.27** | -.30** |
| 4. Stimulation | -.12* | .05 | .24** | **.82**** | .40** | .28** | .07 | -.43** | -.47** | -.35** |
| 5. Hedonism | -.09 | .01 | .21** | .49** | **.75**** | .16** | .05 | -.31** | -.38** | -.37** |
| 6. Achievement | -.09 | -.14** | .04 | .23** | .10 | **.75**** | .27** | -.16** | -.27** | -.38** |
| 7. Power | -.27** | -.37** | -.08 | .05 | .06 | .42** | **.66**** | .04 | -.24** | -.28** |
| 8. Security | .02 | -.20** | -.17** | -.39** | -.20** | -.21** | -.07 | **.67**** | .20** | .24** |
| 9. Conformity | -.01 | -.25** | -.40** | -.49** | -.31** | -.19** | -.08 | .24** | **.76**** | .39** |
| 10. Tradition | .02 | -.37** | -.39** | -.27** | -.20** | -.17** | -.04 | .29** | .30** | **.80**** |

*Notes*. *N* = 363. * *p* < .05, ** *p* < .01.

**Table 26S**

*Pairwise Comparisons Between Ipsatized Personal and Others’ Values in Three Value Priority Groups (High, Middle, Low) in Study 2 (USA)*

| Value | Sample rank | Low Priority Group | | | | Middle Priority Group | | | | High Priority Group | | | | Interaction effect  *F*(2, 360) | η^2^_partial_ |
| --- | --- | --- | --- | --- | --- | --- | --- | --- | --- | --- | --- | --- | --- | --- | --- |
|  |  | Mean Diff. | *SE* | *n* | *d* | Mean Diff. | *SE* | *n* | *d* | Mean Diff. | *SE* | *n* | *d* |  |  |
| Benevolence | 2 | -0.18*** | 0.05 | 119 | **-0.35** | 0.34*** | 0.04 | 124 | **0.81** | 0.80*** | 0.05 | 120 | **1.34** | 106.87*** | .373 |
| Universalism | 3 | 0.23*** | 0.06 | 121 | **0.38** | 0.74*** | 0.05 | 122 | **1.25** | 1.63*** | 0.07 | 120 | **2.10** | 136.70*** | .432 |
| Self-direction | 1 | -0.08^a^ | 0.05 | 120 | **-0.15** | 0.46*** | 0.05 | 123 | **0.79** | 1.33*** | 0.08 | 120 | **1.48** | 127.45*** | .415 |
| Stimulation | 8 | -1.16*** | 0.08 | 123 | **-1.34** | -0.10^a^ | 0.06 | 120 | **-0.15** | 0.96*** | 0.09 | 120 | **1.03** | 197.04*** | .523 |
| Hedonism | 5 | -1.21*** | 0.09 | 120 | **-1.21** | -0.22*** | 0.06 | 124 | **-0.35** | 0.43*** | 0.08 | 119 | **0.52** | 117.06*** | .394 |
| Achievement | 6 | -1.40*** | 0.07 | 121 | **-1.71** | -0.51*** | 0.06 | 123 | **-0.83** | 0.14* | 0.06 | 119 | **0.20** | 139.60*** | .437 |
| Power | 10 | -2.10*** | 0.10 | 121 | **-1.88** | -1.47*** | 0.09 | 122 | **-1.48** | -0.36*** | 0.07 | 120 | **-0.49** | 100.64*** | .359 |
| Security | 4 | -0.82*** | 0.06 | 121 | **-1.28** | -0.14** | 0.04 | 121 | **-0.30** | 0.19*** | 0.05 | 121 | **0.33** | 98.54*** | .354 |
| Conformity | 7 | -0.61*** | 0.07 | 122 | **-0.78** | 0.31*** | 0.05 | 120 | **0.54** | 1.00*** | 0.07 | 121 | **1.23** | 147.54*** | .450 |
| Tradition | 9 | -0.79*** | 0.05 | 120 | **-1.44** | 0.13* | 0.05 | 122 | **0.22** | 0.65*** | 0.07 | 121 | **0.83** | 152.20*** | .458 |

*Notes*. *N* = 363. Mean Difference is calculated as personal values minus others’ values. ^a^ *p* < .10, * *p* < .05, ** *p* < .01, *** *p* < .001.

**Table 27S**

*Means, Standard Deviations, and Ranks of Ipsatized Personal and Others’ Values in Study 3 (Israel)*

| Value | Personal | | | | Others | | | |
| --- | --- | --- | --- | --- | --- | --- | --- | --- |
|  | Rank | *M* | *SD* | Cronbach’s alpha | Rank | *M* | *SD* | Cronbach’s alpha |
| Benevolence | 2 | 0.57 | 0.65 | .73 | 8 | -0.11 | 0.64 | .77 |
| Universalism | 5 | 0.13 | 0.64 | .73 | 6 | -0.06 | 0.63 | .81 |
| Self-direction | 1 | 0.65 | 0.58 | .64 | 2 | 0.53 | 0.60 | .70 |
| Stimulation | 8 | -0.27 | 1.03 | .78 | 7 | -0.07 | 0.92 | .70 |
| Hedonism | 7 | -0.09 | 1.19 | .60 | 3 | 0.34 | 1.07 | .46 |
| Achievement | 3 | 0.32 | 0.71 | .72 | 1 | 0.74 | 0.82 | .74 |
| Power | 10 | -1.19 | 1.14 | .67 | 5 | 0.08 | 1.20 | .59 |
| Security | 4 | 0.31 | 0.70 | .74 | 4 | 0.16 | 0.65 | .66 |
| Conformity | 6 | -0.02 | 0.75 | .75 | 9 | -0.33 | 0.70 | .70 |
| Tradition | 9 | -1.08 | 1.02 | .69 | 10 | -0.96 | 0.73 | .73 |

*Note*. *N* = 227.

**Table 28S**

*Correlations Between Ipsatized Personal and Others’ Values in Study 3 (Israel)*

| Personal values | Others’ values | | | | | | | | | |
| --- | --- | --- | --- | --- | --- | --- | --- | --- | --- | --- |
|  | 1. | 2. | 3. | 4. | 5. | 6. | 7. | 8. | 9. | 10. |
| 1. Benevolence | **.25**** | -.04 | .12 | .02 | -.17** | .13* | .00 | -.03 | -.09 | -.19** |
| 2. Universalism | .04 | **.15*** | .00 | -.02 | -.04 | .02 | -.14* | -.08 | -.10 | .07 |
| 3. Self-direction | .01 | -.05 | **.12** | -.08 | .00 | .12 | .01 | .00 | -.12 | -.02 |
| 4. Stimulation | -.06 | .02 | -.14* | **-.04** | .02 | -.13* | -.02 | .05 | .18** | .11 |
| 5. Hedonism | -.04 | -.02 | -.07 | -.11 | **.36**** | .00 | -.04 | .03 | .00 | .00 |
| 6. Achievement | .06 | -.05 | .08 | .04 | -.12 | **.30**** | -.01 | -.19** | -.05 | -.07 |
| 7. Power | .01 | -.22** | -.19** | .03 | .11 | .04 | **.36**** | -.01 | .02 | -.02 |
| 8. Security | -.09 | -.02 | -.03 | .01 | .10 | -.11 | .07 | **.27**** | -.01 | -.13 |
| 9. Conformity | -.13* | -.09 | .20** | .02 | -.07 | -.07 | .10 | -.04 | **.17*** | -.02 |
| 10. Tradition | -.05 | .15* | -.04 | .07 | -.07 | -.17** | -.17** | .00 | .04 | **.15*** |

*Notes*. *N* = 227. * *p* < .05, ** *p* < .01.

**Table 29S**

*Correlations Between Ipsatized Personal Values and Self-Others’ Differences in Study 3 (Israel)*

| Personal values | Self-others’ differences | | | | | | | | | |
| --- | --- | --- | --- | --- | --- | --- | --- | --- | --- | --- |
|  | 1. | 2. | 3. | 4. | 5. | 6. | 7. | 8. | 9. | 10. |
| 1. Benevolence | **.62**** | .05 | -.17* | -.29** | -.17* | -.17** | -.31** | -.05 | .15* | .18** |
| 2. Universalism | -.01 | **.66**** | .12 | .00 | .02 | -.27** | -.25** | -.33** | -.21** | -.12 |
| 3. Self-direction | -.09 | .15* | **.65**** | .23** | .15* | -.02 | -.05 | -.29** | -.20** | -.41** |
| 4. Stimulation | -.26** | -.03 | .29** | **.76**** | .34** | .11 | .14* | -.26** | -.38** | -.42** |
| 5. Hedonism | -.25** | .01 | .17** | .35** | **.63**** | .02 | .26** | -.10 | -.29** | -.42** |
| 6. Achievement | -.11 | -.22** | .03 | -.04 | .12 | **.51**** | .29** | .07 | -.09 | -.23** |
| 7. Power | -.31** | -.18** | .11 | .08 | .14* | .22** | **.54**** | .08 | -.10 | -.29** |
| 8. Security | .00 | -.34** | -.22** | -.19** | -.16* | .02 | .01 | **.64**** | .22** | .14* |
| 9. Conformity | .18** | -.20** | -.42** | -.23** | -.27** | -.07 | -.18** | .25** | **.68**** | .23** |
| 10. Tradition | .09 | -.18** | -.32** | -.34** | -.39** | -.09 | -.14* | .05 | .17* | **.78**** |

*Notes*. *N* = 227. * *p* < .05, ** *p* < .01.

**Table 30S**

*Pairwise Comparisons Between Ipsatized Personal and Others’ Values in the Three Value Priority Groups (High, Middle, Low) in Study 3 (Israel)*

| Value | Sample rank | Low Priority Group | | | | Middle Priority Group | | | | High Priority Group | | | | Interaction effect  *F*(2, 224) | η^2^_partial_ |
| --- | --- | --- | --- | --- | --- | --- | --- | --- | --- | --- | --- | --- | --- | --- | --- |
|  |  | Mean Diff. | *SE* | *n* | *d* | Mean Diff. | *SE* | *n* | *d* | Mean Diff. | *SE* | *n* | *d* |  |  |
| Benevolence | 2 | 0.15* | 0.07 | 75 | **0.23** | 0.67*** | 0.06 | 77 | **1.21** | 1.22*** | 0.09 | 75 | **1.56** | 49.44*** | .306 |
| Universalism | 5 | -0.42*** | 0.08 | 75 | **-0.63** | 0.26** | 0.09 | 76 | **0.37** | 0.73*** | 0.08 | 76 | **1.10** | 55.05*** | .330 |
| Self-direction | 1 | -0.47*** | 0.07 | 75 | **-0.74** | 0.18* | 0.07 | 76 | **0.30** | 1.03*** | 0.07 | 76 | **1.03** | 61.84*** | .356 |
| Stimulation | 8 | -1.43*** | 0.13 | 75 | **-1.31** | -0.07 | 0.12 | 76 | **-0.07** | 0.89*** | 0.12 | 76 | **0.88** | 93.88*** | .456 |
| Hedonism | 7 | -1.36*** | 0.13 | 75 | **-1.20** | -0.31** | 0.12 | 76 | **-0.30** | 0.37** | 0.12 | 76 | **0.36** | 50.43*** | .310 |
| Achievement | 3 | -0.94*** | 0.09 | 76 | **-1.25** | -0.33*** | 0.09 | 76 | **-0.43** | 0.01 | 0.11 | 75 | **0.02** | 26.14*** | .189 |
| Power | 10 | -2.00*** | 0.14 | 76 | **-1.64** | -1.32*** | 0.14 | 75 | **-1.08** | -0.48*** | 0.12 | 76 | **-0.45** | 31.96*** | .222 |
| Security | 4 | -0.42*** | 0.09 | 75 | **-0.53** | 0.20** | 0.06 | 76 | **0.36** | 0.66*** | 0.08 | 76 | **0.98** | 47.64*** | .298 |
| Conformity | 6 | -0.35*** | 0.08 | 75 | **-0.49** | 0.31*** | 0.07 | 78 | **0.48** | 0.99*** | 0.11 | 74 | **1.05** | 55.41*** | .331 |
| Tradition | 9 | -1.17*** | 0.08 | 75 | **-1.60** | -0.10 | 0.09 | 76 | **-0.12** | 0.88*** | 0.10 | 76 | **0.98** | 120.45*** | .518 |

*Notes*. *N* = 227. Mean Difference is calculated as personal values minus others’ values. * *p* < .05, ** *p* < .01, *** *p* < .001.
